# Publisher Correction: Oligomeric scaffolding for curvature generation by ER tubule-forming proteins

**DOI:** 10.1038/s41467-023-39182-1

**Published:** 2023-06-13

**Authors:** Yun Xiang, Rui Lyu, Junjie Hu

**Affiliations:** 1 National Laboratory of Biomacromolecules, CAS Center for Excellence in Biomacromolecules, Institute of Biophysics, Chinese Academy of Sciences, Beijing, 100101 China; 2 University of Chinese Academy of Sciences, Beijing, 100101 China

**Keywords:** Endoplasmic reticulum, Membrane structure and assembly, Structural biology

Correction to: *Nature Communications* 10.1038/s41467-023-38294-y, published online 05 May 2023

The Source Data file and the Reporting Summary were missing from this article and have now been uploaded. The original article has been corrected.

## Reporting summary

Further information on research design is available in the Nature Portfolio Reporting Summary linked to this article.

## Supplementary information

Reporting Summary

## Source data

Source Data

