# Supplementary material for: Oligomeric scaffolding for curvature generation by ER tubule-forming proteins
Source: Nat Commun. 2023 May 5;14:2617. doi: 10.1038/s41467-023-38294-y (PMC10162974; doi:10.1038/s41467-023-38294-y)
Supplement: Supplementary file 1 — Supplementary Information [file 41467_2023_38294_MOESM1_ESM.pdf]

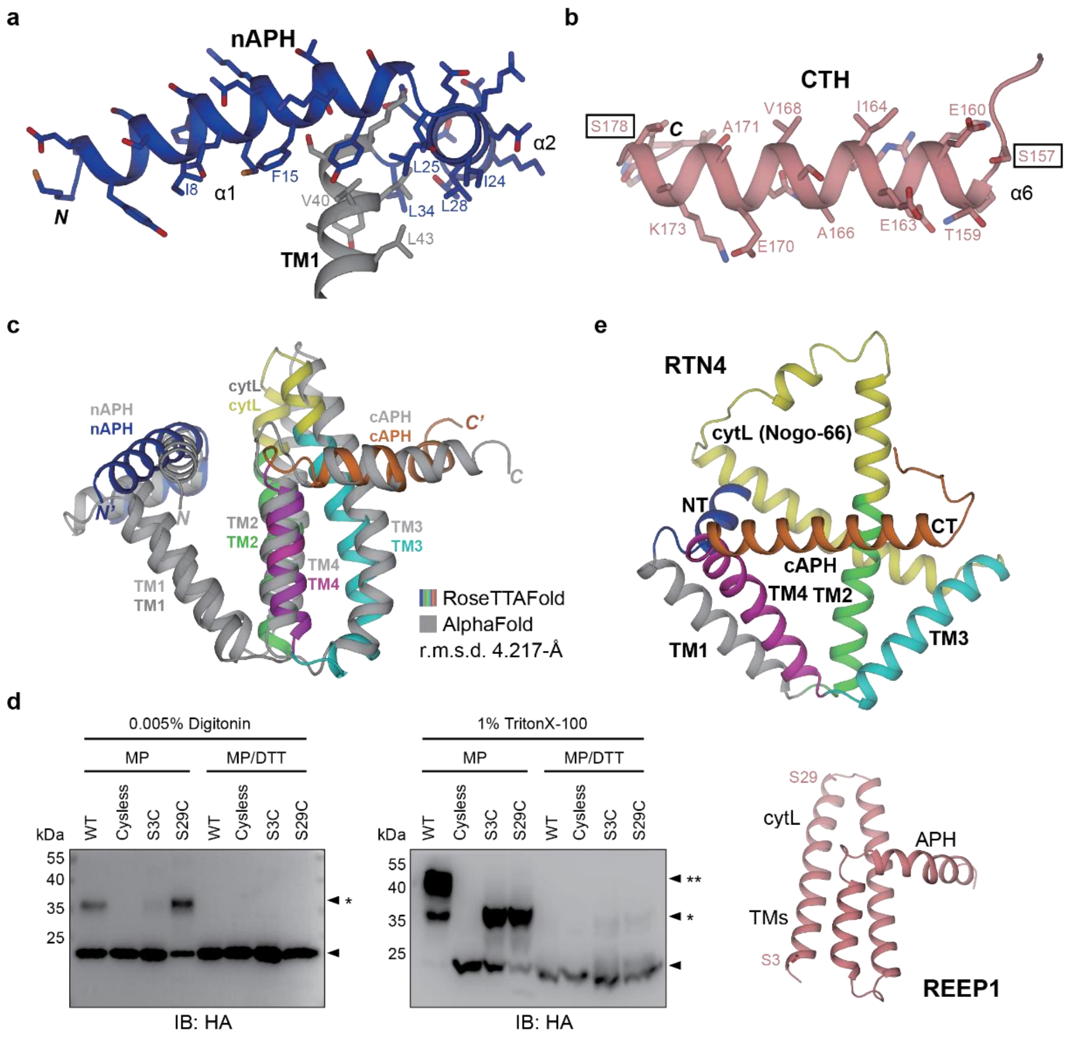

Supplementary Fig. 1 Structural comparison of REEP and RTN

(**a**) Structural details of the N-terminal amphipathic helices (nAPHs). Domains are colored as in **Fig. 1b** and side chains of key residues are shown as sticks. Residues in the nAPH exhibit an amphipathic pattern, with hydrophobic residues pointing towards the membrane and hydrophilic residues pointing in the opposite direction. The hydrophobic face of the nAPH is engaged by residues from TM1. (**b**) As in (**a**), but with C-terminal helix (CTH). Notably, the helix also has an amphipathic pattern, which likely forms coiled coil interactions. Residues used in Cys-cross-linking are boxed. (**c**) Structural comparison of Yop1p predicted by RoseTTAFold and AlphaFold. RF-predicted Yop1p is shown as in **Fig. 1b** and AF-predicted in gray. Major domains are labeled. The root mean squared deviation (RMSD) measurements are indicated. (**d**) Topology analysis of REEP1. Single cysteines were placed at the indicated positions into cysless REEP1. Wild-type (WT) REEP1, which contains two cysteines, was used for comparison. HA-tagged REEP1 mutants or WT were expressed in COS-7 cells. Digitonin or Triton was added, followed by maleimide PEG 5 kDa (MP) in the absence or presence of DTT. Samples were separated by non-reducing SDS-PAGE and immunoblotted (IB) by anti-HA antibodies. Unmodified REEP1 is indicated by arrowheads. Single modification is indicated by one asterisk (*) and double modification by two asterisks (**). The predicted structure of REEP1 is shown on the right with mutated residues highlighted as sticks. (**e**) Structure of the core RTN4 predicted by AlphaFold. Regions of RTN4 are shown in cartoon representation and colored as in Yop1p. cAPH, C-terminal amphipathic helix; TM, transmembrane; cytL, cytosolic loop, also known as Nogo-66. Source data are provided as a Source Data file.

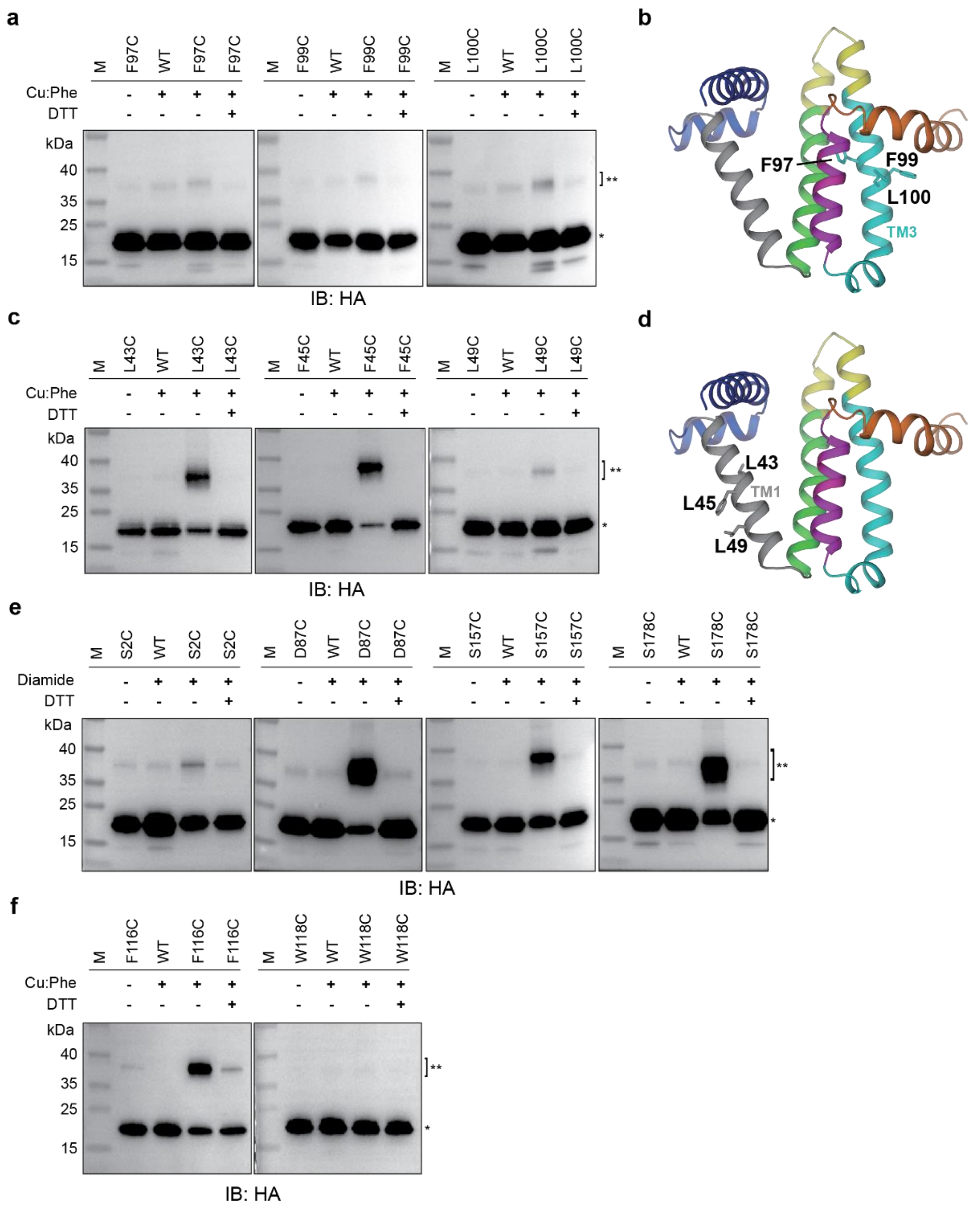

Supplementary Fig. 2 Additional cysteine cross-linking of Yop1p

(**a**) Yop1p dimerization probed by Cys-cross-linking. Membrane fractions of yeast cells expressing HA-tagged Yop1p, including wild-type (WT) or Cys mutants in TM3, were treated with 1 mM copper-o-phenanthroline (Cu:Phe). Reduction by DTT was used as a control. Samples were separated by non-reducing SDS-PAGE and immunoblotted (IB) by anti-HA antibodies. A single asterisk (*) indicates the monomer and double asterisks (**) the dimer. M, molecular marker. (**b**) Positions of Cys-replaced residues used in (**a**). (**c**) As in (**a**), but with residues in TM1. (**d**) Positions of Cys-replaced residues used in (**c**). (**e**) As in (**a**), but oxidized by 2 mM diamide and with residues in cytosolic regions. (**f**) As in (**a**), but with residues in TM4. Source data are provided as a Source Data file.

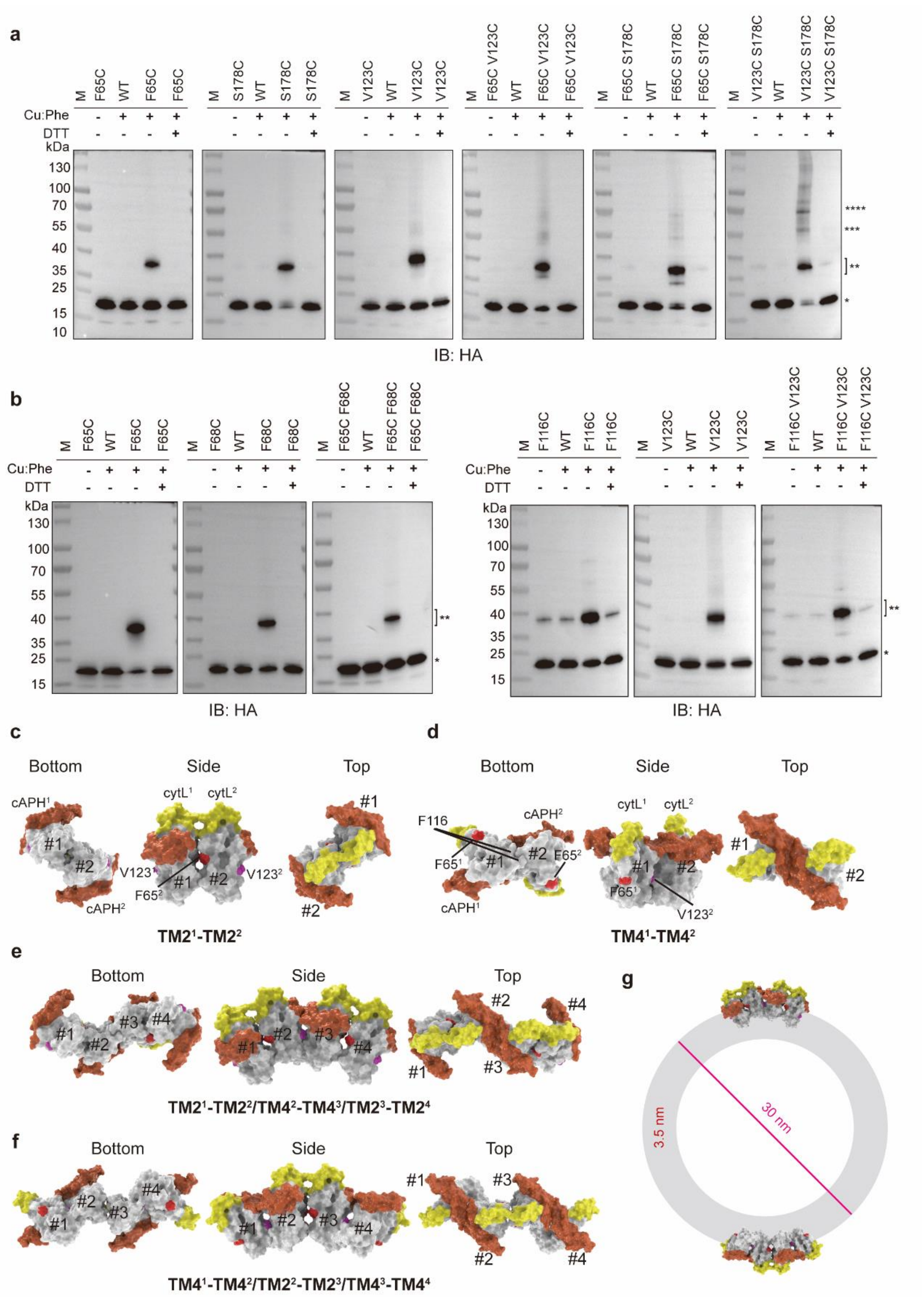

Supplementary Fig. 3 Additional verification of oligomeric scaffolding

(**a**)Yop1p dimerization and oligomerization probed by Cys-cross-linking. Membrane fractions of yeast cells expressing HA-tagged Yop1p, including wild-type (WT) or double Cys mutants, were

treated with 1 mM copper-o-phenanthroline (Cu:Phe). Reduction by DTT was used as a control. Samples were separated by non-reducing SDS-PAGE and immunoblotted (IB) by anti-HA antibodies. The cross-linking of three single mutants are shown on the left for comparison. A single asterisk (*) indicates the monomer and double asterisks (**) the dimer, (***) the trimer and (****) the tetramer. M, molecular marker. (**b**) As in (**a**), but with another double Cys mutants. The cross-linking of two single mutants are shown on the left for comparison. (**c**) TM2-mediated dimerization of Yop1p ΔNT-TM1. The space-filling model of Yop1p ΔNT-TM1 (residues 57-154) is 3D-printed, manually assembled, scanned, and rendered by Maya software. The cytosolic regions of Yop1p are colored as in **Fig. 1b** and the TM regions are colored white. F65 in TM2 is highlighted in red and V123 in TM4 in magenta. Top, side, and bottom views are shown with molecules numbered. The dimer was assembled with the TM2s facing each other. TM, transmembrane; cytL, cytosolic loop; nAPH, N-terminal amphipathic helix; cAPH, C-terminal amphipathic helix. (**d**) As in (**c**), but with TM4-mediated dimerization. (**e**) As in (**c**), but with TM4-centered tetramerization. The tetramer is built with a TM4-based dimer in the middle, flanked by two TM2-based assemblies on each side. (**f**) As in (**c**), but with TM2-centered tetramerization. The tetramer is built with a TM2-based dimer in the middle, flanked by two TM4-based assemblies on each side. (**g**) The two tetramers of Yop1p ΔNT-TM1 are fitted into a cross-section of a tubule with 30 nm diameter. The membrane is shown as a gray ring with its thickness set to 3.5 nm. Yop1p models are placed proportionally. Source data are provided as a Source Data file.

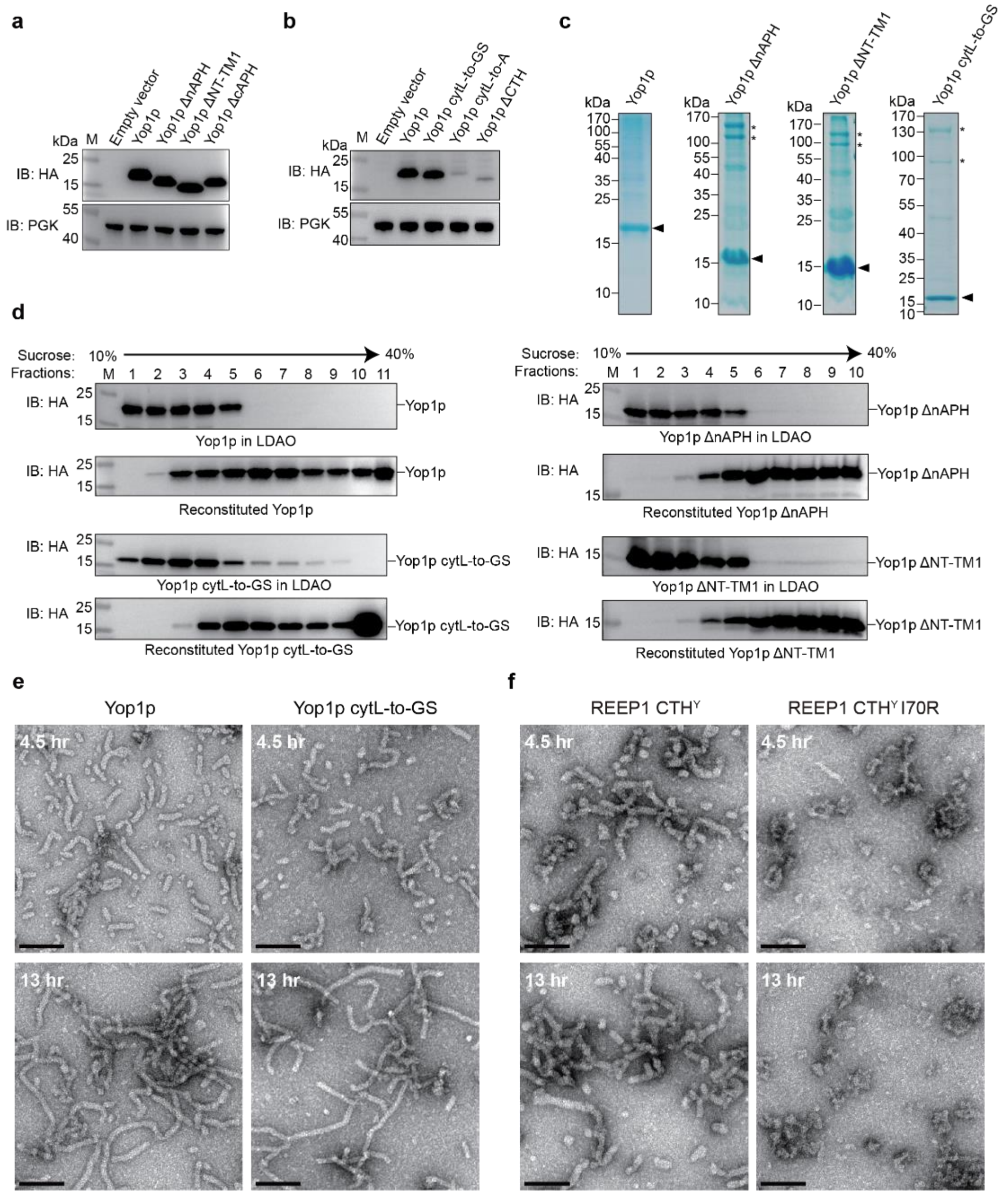

Supplementary Fig. 4 Controls for domain analysis of Yop1p

(**a**) Expression of the indicated Yop1p used in **Fig. 3b**. Empty vector or constructs of HA-tagged Yop1p were transformed into *Δyop1sey1* cells. Lysates were analyzed by immunoblotting (IB) with anti-HA antibodies. PGK was used as a loading control. (**b**) As in (**a**), but with additional Yop1p constructs. (**c**) SDS-PAGE and Coomassie blue staining of purified Yop1p proteins. Arrowheads indicate the Yop1p proteins and asterisks indicate contaminants. (**d**) Sucrose gradient analysis of Yop1p. Detergent-solubilized (1% LDAO) Yop1p-HA (top panels) or reconstituted Yop1p proteoliposomes (bottom panels) were loaded onto a 10%-40% sucrose step gradient, fractionated, and analyzed by IB with anti-HA antibodies. (**e**) Reconstitution of Yop1p wild-type or cytL-to-GS mutant. *E. coli* polar lipids were solubilized with LDAO and mixed with purified Yop1p proteins. The detergent was then removed by incubation with Bio-beads for 4.5 and 13 h. Scale bars, 100 nm. (**f**) as in (**e**), but with reconstitution done by indicated proteins and yeast polar lipids which were solubilized with DDM. Source data are provided as a Source Data file.

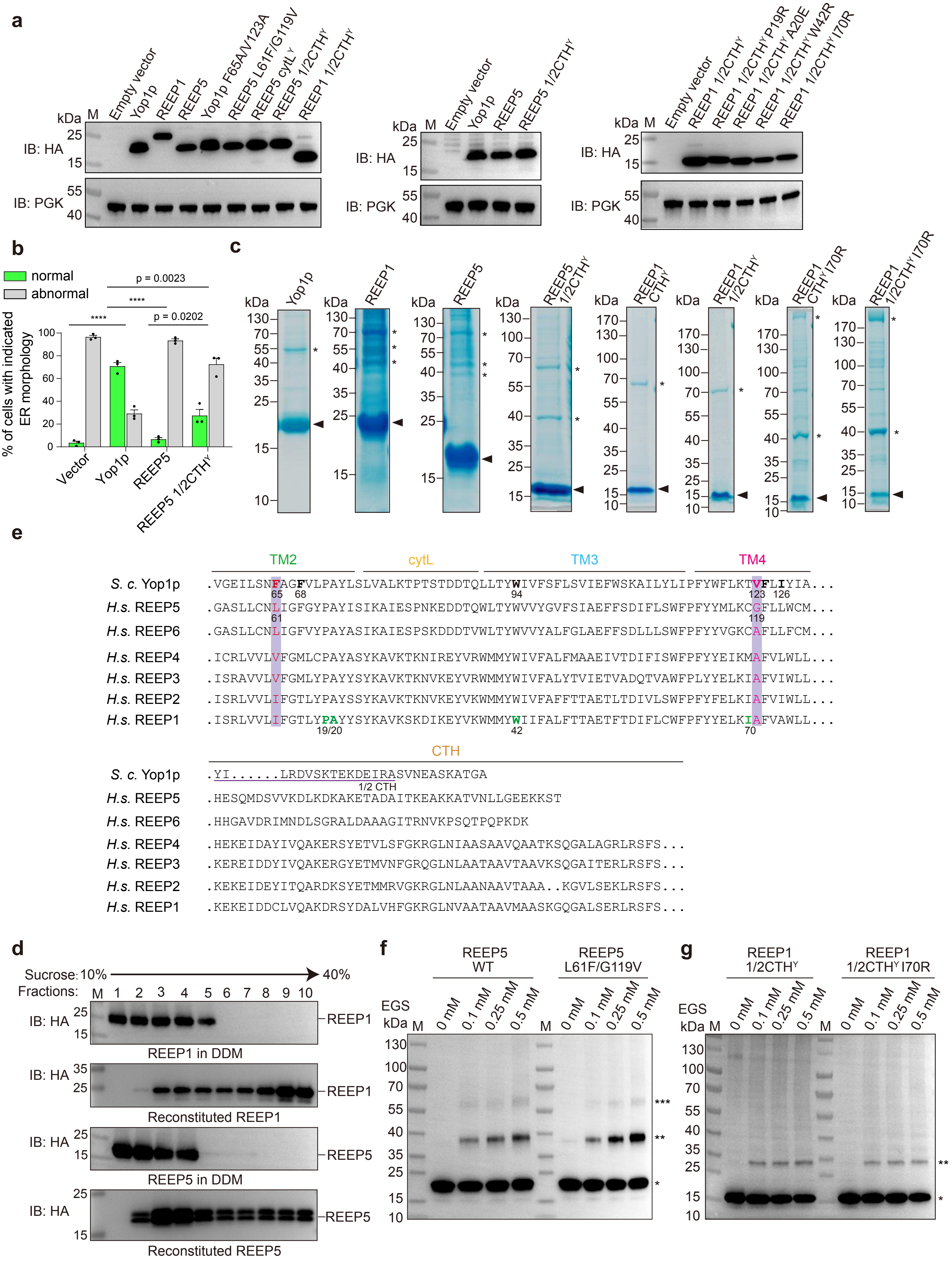
a
kDa M Empty vector Yop1p REEP1 REEP5 Yop1p F65A/V123A REEP5 L61F/G119V REEP5 cytL^Y REEP5 1/2CTH^Y REEP1 1/2CTH^Y
IB: HA
IB: PGK
Empty vector Yop1p REEP5 REEP5 1/2CTH^Y
Empty vector REEP1 1/2CTH^Y REEP1 1/2CTH^Y P19R REEP1 1/2CTH^Y A20E REEP1 1/2CTH^Y W42R REEP1 1/2CTH^Y I70R
b
normal
abnormal
% of cells with indicated ER morphology
p = 0.0023
****
****
p = 0.0202
Vector Yop1p REEP5 REEP5 1/2CTH^Y
c
Yop1p REEP1 REEP5 REEP5 1/2CTH^Y REEP1 CTH^Y REEP1 1/2CTH^Y REEP1 CTH^Y I70R REEP1 1/2CTH^Y I70R
e
TM2 cytL TM3 TM4
S. c. Yop1p .VGEILSNFAGFVLPAYLSLVALKTPTSTDDTQLLTYWIVFSFLSVIEFWSKAILYLIPFYWFLKTVFLIYIA...
65 68 94 123 126
H.s. REEP5 .GASLLCNLIGFGYPAYISIKAIESPNKEDDTQWLTYWVVYGVFSIAEFFSDIFLSWFPFYYMLKCGFLLWCM...
61 119
H.s. REEP6 .GASLLCNLIGFVYPAYASIKAIESPSKDDDTVWLTYWVVYALFGLAEFFSDLLLSWFPFYYVGKCAFLLFCM...
H.s. REEP4 .ICRLVVLVFGMLCPAYASYKAVKTKNIREYVRWMMYWIVFALFMAAEIVTDIFISWFPFYYEIKMAFVLWLL...
H.s. REEP3 .ISRAVVLVFGMLYPAYYSYKAVKTKNVKEYVRWMMYWIVFALYTVIETVADQTVAWFPLYYELKIAFVIWLL...
H.s. REEP2 .ISRLVVLIFGTLYPAYSSYKAVKTKNVKEYVKWMMYWIVFAFFTTAETLTDIVLSWFPFYFELKIAFVIWLL...
H.s. REEP1 .ISRLVVLIFGTLYPAYYSYKAVKSKDIKEYVKWMMYWIIFALFTTAETFTDIFLCWFPFYYELKIAFVAWLL...
19/20 42 70
CTH
S. c. Yop1p .YI......LRDVSKTEKDEIRASVNEASKATGA
1/2 CTH
H.s. REEP5 .HESQMDSVVKDLKDKAKETADAITKEAKKATVNLLGEEKKST
H.s. REEP6 .HHGAVDRIMNDLSGRALDAAAGITRNVKPSQTPQPKDK
H.s. REEP4 .HEKEIDAYIVQAKERSYETVLSFGKRGLNIAASAAVQAATKSQGALAGRLRSFS...
H.s. REEP3 .KEREIDDYIVQAKERGYETMVNFGRQGLNLAATAAVTAAVKSQGAITERLRSFS...
H.s. REEP2 .KEKEIDEYITQARDKSYETMMRVGKRGLNLAANAAVTAAA..KGVLSEKLRSFS...
H.s. REEP1 .KEKEIDDCLVQAKDRSYDALVHFGKRGLNVAATAAVMAASKGQGALSERLRSFS...
d
Sucrose: 10% 40%
Fractions: M 1 2 3 4 5 6 7 8 9 10
IB: HA
REEP1
REEP1 in DDM
Reconstituted REEP1
REEP5
REEP5 in DDM
Reconstituted REEP5
f
REEP5 WT
REEP5 L61F/G119V
EGS
0 mM 0.1 mM 0.25 mM 0.5 mM
g
REEP1 1/2CTH^Y
REEP1 1/2CTH^Y I70R

Supplementary Fig. 5 Additional comparison of REEP proteins

(**a**) Expression of the indicated REEP used in **Fig. 4b,e** and (**b**). Empty vector or constructs of HA-tagged REEP were transformed into *Δyop1/sey1* or *Δrtn1/rtn2/yop1* cells. Lysates were analyzed by immunoblotting (IB) with anti-HA antibodies. PGK was used as a loading control. (**b**) ER morphology rescued by the REEP proteins. Sec63p-GFP was expressed in cells lacking Rtn1p, Rtn2p, and Yop1p (*Δrtn1/rtn2/yop1*). Vector or indicated REEP constructs were also transformed into these cells. The ER was visualized and categorized by counting at least 100 cells per sample. The quantitative data are shown as mean ± SEM of three repeats. NS, not significant; ****, $p < 0.00001$, unpaired two-sided Student's t-test. (**c**) SDS-PAGE and Coomassie blue staining of purified REEPs. Arrowheads indicate the desired proteins and asterisks (*) indicate contaminants. (**d**) Sucrose gradient analysis of REEP. Detergent-solubilized (1% DDM) REEP1/5-HA (top panels) or reconstituted REEP1/5 proteoliposomes (bottom panels) were loaded onto a 10%-40% sucrose step gradient, fractionated, and analyzed by IB with anti-HA antibodies. (**e**) Alignment of REEP proteins. Key residues for dimerization (bold) and SPG31 mutations (green) are numbered and highlighted in bold. The F65 in TM2 and V123 in TM4 are boxed for comparison. *S.c., Saccharomyces cerevisiae; H.s., Homo sapiens.* (**f**) EGS cross-linking of REEP5. Isolated membranes from cells expressing REEP5, wild-type or L61F/G119V mutant, were treated with increasing concentrations of EGS and analyzed by SDS-PAGE, and oligomers (as indicated by asterisks) were visualized by IB with anti-HA antibody. (**g**) As in (**f**), but with REEP1 1/2CTH^{Y}, wild-type or I70R mutant. Source data are provided as a Source Data file.

Supplementary Table 1: Plasmids used in this study

Plasmids Description	References
pWP1098-Sec63p-GFP	This Study
pYC2 CT 300Yop1p(WT)-HA	This Study
pYC2 CT 300Yop1p(S2C)-HA	This Study
pYC2 CT 300Yop1p(L43C)-HA	This Study
pYC2 CT 300Yop1p(F45C)-HA	This Study
pYC2 CT 300Yop1p(L49C)-HA	This Study
pYC2 CT 300Yop1p(F65C)-HA	This Study
pYC2 CT 300Yop1p(F68C)-HA	This Study
pYC2 CT 300Yop1p(D87C)-HA	This Study
pYC2 CT 300Yop1p(L90C)-HA	This Study
pYC2 CT 300Yop1p(L91C)-HA	This Study
pYC2 CT 300Yop1p(W94C)-HA	This Study
pYC2 CT 300Yop1p(F97C)-HA	This Study
pYC2 CT 300Yop1p(F99C)-HA	This Study
pYC2 CT 300Yop1p(L100C)-HA	This Study
pYC2 CT 300Yop1p(F116C)-HA	This Study
pYC2 CT 300Yop1p(W118C)-HA	This Study
pYC2 CT 300Yop1p(V123C)-HA	This Study
pYC2 CT 300Yop1p(F124C)-HA	This Study
pYC2 CT 300Yop1p(L125C)-HA	This Study
pYC2 CT 300Yop1p(I126C)-HA	This Study
pYC2 CT 300Yop1p(S157C)-HA	This Study
pYC2 CT 300Yop1p(S178C)-HA	This Study
pYC2 CT 300Yop1p(F65C/F68C)-HA	This Study
pYC2 CT 300Yop1p(F65C/V123C)-HA	This Study
pYC2 CT 300Yop1p(F65C/S178C)-HA	This Study
pYC2 CT 300Yop1p(V123C/S178C)-HA	This Study
pYC2 CT 300Yop1p(F65A/V123A)-HA	This Study
pYC2 CT 300Yop1p(cytL-to-GS)-HA	This Study
pYC2 CT 300Yop1p(cytL-to-A)-HA	This Study
pYC2 CT 300Yop1p∆nAPH-HA	This Study
pESC-URA-dGAL300Yop1p∆NT-TM1-HA	This Study
pYC2 CT 300Yop1p∆cAPH-HA	This Study
pESC-URA-dGAL300Yop1p∆CTH-HA	This Study
pESC-URA-500prodGAL-REEP1-HA-500ter	This Study
pESC-URA-500prodGAL-REEP1 1/2CTH^{Y}-HA-500ter	This Study
pESC-URA-500prodGAL-REEP1 1/2CTH^{Y}(P19R)-HA-500ter	This Study
pESC-URA-500prodGAL-REEP1 1/2CTH^{Y}(A20E)-HA-500ter	This Study
pESC-URA-500prodGAL-REEP1 1/2CTH^{Y}(W42R)-HA-500ter	This Study
pESC-URA-500prodGAL-REEP1 1/2CTH^{Y}(I70R)-HA-500ter	This Study
pESC-URA-500prodGAL-REEP5-HA-500ter	This Study

pESC-URA-500prodGAL-REEP5(L61F/G119V)-HA-500ter	This Study
pESC-URA-500prodGAL-REEP5 1/2CTH^{Y}-HA-500ter	This Study
pESC-URA-500prodGAL-REEP5 cytL^{Y-}HA-500ter	This Study
pESC-URA-His$_{6}$-Yop1p-HA	This Study
pESC-URA-Yop1p-HA-StrepII	This Study
pESC-URA-Yop1pΔnAPH-HA-StrepII	This Study
pESC-URA-Yop1pΔNT-TM1-HA-StrepII	This Study
pESC-URA-Yop1p(cytL-to-GS)-HA-StrepII	This Study
pcDNA4/TO-CMV-REEP1-HA-StrepII	This Study
pcDNA4/TO-CMV-REEP1 1/2CTH^{Y}-HA-StrepII	This Study
pcDNA4/TO-CMV-REEP1 CTH^{Y}-HA-StrepII	This Study
pcDNA4/TO-CMV-REEP1 1/2CTH^{Y} (I70R)-HA-StrepII	This Study
pcDNA4/TO-CMV-REEP1 CTH^{Y} (I70R)-HA-StrepII	This Study
pcDNA4/TO-CMV-REEP1(WT)-HA	This Study
pcDNA4/TO-CMV-REEP1(S3C)-HA	This Study
pcDNA4/TO-CMV-REEP1(S29C)-HA	This Study
pcDNA4/TO-CMV-REEP5-HA-StrepII	This Study
pcDNA4/TO-CMV-REEP5 1/2CTH^{Y}-HA-StrepII	This Study

Supplementary Table 2: Primers used in this Study.	
**Primer name**	**Primer sequence (5'-3')**
Yop1p (S2C) F	ACTCCAATCATGTGCGAATATGCATCTAGTATTCACTCTCAAATGAAACA
Yop1p (S2C) R	AGATGCATATTCGCACATGATTGGAGTGCGGTTATGTC
Yop1p (L43C) F	TTAGTTGCTGGTTGCGGTTTCGCTTATCTCCTTTTGATTTTTATTAACG
Yop1p (L43C) R	ATAAGCGAAACCGCAACCAGCAACTAAATAAGATTTAGGCAAATTAGT
Yop1p (F45C) F	TTTAGGTTGCGCTTATCTCCTTTTGATTTTTATTAACGTGGGAG
Yop1p (F45C) R	GGAGATAAGCGCAACCTAAACCAGCAACTAAATAAGATTTAGGC
Yop1p (L49C) F	TTCGCTTATCTCTGCTTGATTTTTATTAACGTGGGAGGTGTAGG
Yop1p (L49C) R	AATAAAAATCAAGCAGAGATAAGCGAAACCTAAACCAGC
Yop1p (F65C) F	ATTCTTTCCAATTGCGCTGGGTTTGTGTTGCCAG
Yop1p (F65C) R	CACAAACCCAGCGCAATTGGAAAGAATTTCACCTACACCTCC
Yop1p (F68C) F	AATTTTGCTGGGTGCGTGTTGCCAGCATATTTATCGTTGG
Yop1p (F68C) R	TGGCAACACGCACCCAGCAAAATTGGAAAGAATTTCAC
Yop1p (D87C) F	ACGTCCACCGATTGCACACAACTCTTGACCTACTGGATT
Yop1p (D87C) R	CAAGAGTTGTGTGCAATCGGTGGACGTTGGTGT
Yop1p (L90C) F	ATGACACACAATGCTTGACCTACTGGATTGTCTTTTCATTTTTGAG
Yop1p (L90C) R	GGTCAAGCATTGTGTGTCATCGGTGGACG
Yop1p (L91C) F	GACACACAACTCTGCACCTACTGGATTGTCTTTTCATTTTTGAGT
Yop1p (L91C) R	AGTAGGTGCAGAGTTGTGTGTCATCGGTGGAC
Yop1p (W94C) F	CTCTTGACCTACTGCATTGTCTTTTCATTTTTGAGTGTCATTGAATTCT
Yop1p (W94C) R	TGAAAAGACAATGCAGTAGGTCAAGAGTTGTGTGTCATCG
Yop1p (F97C) F	TACTGGATTGTCTGCTCATTTTTGAGTGTCATTGAATTCTGGTCC
Yop1p (F97C) R	ACTCAAAAATGAGCAGACAATCCAGTAGGTCAAGAGTTGTG
Yop1p (F99C) F	CTTTTCATGCTTGAGTGTCATTGAATTCTGGTCCAAG
Yop1p (F99C) R	TGACACTCAAGCATGAAAAGACAATCCAGTAGGTCAAGAGTT
Yop1p (L100C) F	GTCTTTTCATTTTGCAGTGTCATTGAATTCTGGTCCAAGG
Yop1p (L100C) R	TTCAATGACACTGCAAAATGAAAAGACAATCCAGTAGGTCAAGAG
Yop1p (F116C) F	TATTTGATTCCATGCTACTGGTTTTTGAAAACCGTTTTCTTAATCTACAT
Yop1p (F116C) R	CCAGTAGCATGGAATCAAATATAGAATTGCCTTGGACCA
Yop1p (W118C) F	ATTCCATTCTACTGCTTTTTGAAAACCGTTTTCTTAATCTACATTGCC
Yop1p (W118C) R	CGGTTTTCAAAAAGCAGTAGAATGGAATCAAATATAGAATTGCCTTGG
Yop1p (V123C) F	TTTTTGAAAACCTGCTTCTTAATCTACATTGCCTTGCCTCAAAC
Yop1p (V123C) R	GTAGATTAAGAAGCAGGTTTTCAAAAACCAGTAGAATGGAATCAAAT
Yop1p (F124C) F	TTGAAAACCGTTTGCTTAATCTACATTGCCTTGCCTCAAACTG
Yop1p (F124C) R	GCAATGTAGATTAAGCAAACGGTTTTCAAAAACCAGTAGAATGG
Yop1p (L125C) F	AAAACCGTTTTCTGCATCTACATTGCCTTGCCTCAAAC
Yop1p (L125C) R	GGCAATGTAGATGCAGAAAACGGTTTTCAAAAACCAGTAGAATG
Yop1p (I126C) F	ACCGTTTTCTTATGCTACATTGCCTTGCCTCAAACTGG
Yop1p (I126C) R	CAAGGCAATGTAGCATAAGAAAACGGTTTTCAAAAACCAGTAGAATGG
Yop1p (S157C) F	AGAGATGTTTGCAAGACAGAAAAGGATGAAATTAGAGCTTCC
Yop1p (S157C) R	TCTGTCTTGCAAACATCTCTTAGGATATATCTGTCGGTCAAT

Yop1p (S178C) F	GCTACAGGTGCTTGCGTTCATTACCCATACGATGTTCCAGAT
Yop1p (S178C) R	TGGGTAATGAACGCAAGCACCTGTAGCCTTAGAAGC
Yop1p (F65C/F68C) F	AATTGCGCTGGGTGCGTGTTGCCAGCATATTTATCGTTGG
Yop1p (F65C/F68C) R	TGCTGGCAACACGCACCCAGCGCAATTGGAAAGAAT
Yop1p (F65A) F	ATTCTTTCCAATGCTGCTGGGTTTGTGTTGCCAG
Yop1p (F65A) R	CACAAACCCAGCAGCATTGGAAAGAATTTCACCTACACCTCC
Yop1p (V123A) F	TTTTTGAAAACCGCTTTCTTAATCTACATTGCCTTGCCTCAAAC
Yop1p (V123A) R	GTAGATTAAGAAAGCGGTTTTCAAAAACCAGTAGAATGGAATCAAAT
Yop1p (cytL-to-GS) F	GGGAGTGGAAGCAGCCAACTCTTGACCTACTGGATTGTCT
Yop1p (cytL-to-GS) R	GGCTGCTTCCACTCCCAGCAACCAACGATAAATATGCTGG
Yop1p (cytL-to-A) F	AGCCGCAGCCGCGGCCGCCGCCGCGCAACTCTTGACCTACTGGATTGTCT
Yop1p (cytL-to-A) R	CGGCGGCCGCGGCTGCGGCTGCGGCAGCAACCAACGATAAATATGCTGG
Yop1pΔnAPH F	TCCAATCATGTTGCCTAAATCTTATTTAGTTGCTGGTTTAGG
Yop1pΔnAPH R	ATTTAGGCAACATGATTGGAGTGCGGTTATGTC
pESC-URA-dGAL 300Yop1pΔNT-TM1 F	TCCAATCATGGGAGGTGTAGGTGAAATTCTTTCCAA
pESC-URA-dGAL 300Yop1pΔNT-TM1 R	CTACACCTCCCATGATTGGAGTGCGGTTATGT
Yop1pΔcAPH F	ATAACCGCACTCCAATCATGTCCGAATATGCATCTAGTATTCACT
Yop1pΔcAPH R	TCTGGAACATCGTATGGGTATCTGTCGGTCAATGGGGCTA
pESC-URA-dGAL 300Yop1pΔCTH F	ATAACCGCACTCCAATCATGTCCGAATATGCATCTAGTATTCACT
pESC-URA-dGAL 300Yop1pΔCTH R	TCTGGAACATCGTATGGGTAAACATCTCTTAGGATATATCTGTCGGTCA
pESC-URA-500prodGAL REEP1 1/2CTH^{Y} F	TGTTAGCAAGACAGAAAAGGATGAAATTAGAGCTTACCCATACGACGTCCCAGACT
pESC-URA-500prodGAL REEP1 1/2CTH^{Y} R	CCTTTTCTGTCTTGCTAACATCTCTTAGGATATAAGAAGACAAAGTTGGATGGACG
pESC-URA-500prodGAL REEP1 1/2CTH^{Y}(P19R) F	GGTACTTTGTATAGGGCTTACTACTCTTACAAAGCTGTCAAGT
pESC-URA-500prodGAL REEP1 1/2CTH^{Y}(P19R) R	AGAGTAGTAAGCCCTATACAAAGTACCGAAAATCAAAACGACCA
pESC-URA-500prodGAL REEP1 1/2CTH^{Y}(A20E) F	ACTTTGTATCCAGAGTACTACTCTTACAAAGCTGTCAAGTCTAAAGAC
pESC-URA-500prodGAL REEP1 1/2CTH^{Y}(A20E) R	GTAAGAGTAGTACTCTGGATACAAAGTACCGAAAATCAAAACGAC
pESC-URA-500prodGAL REEP11/2CTH^{Y}(W42R) F	TGGATGATGTATCGGATTATTTTTGCTTTGTTCACTACTGCTGAG
pESC-URA-500prodGAL REEP11/2CTH^{Y}(W42R) R	AGCAAAAATAATCCGATACATCATCCACTTGACGTATTCCTTAATG
pESC-URA-500prodGAL	TACGAGTTGAAGAGGGCTTTTGTCGCTTGGTTGTTGT

REEP1 1/2CTH^{Y}(I70R) F	
pESC-URA-500prodGAL REEP1 1/2CTH^{Y}(I70R) R	AGCGACAAAAGCCCTCTTCAACTCGTAGTAGAATGGGAACCA
pESC-URA-500prodGAL REEP5(L61F) F	TTGTTGTGTAATTTTATTGGTTTTGGCTACCCCG
pESC-URA-500prodGAL REEP5(L61F) R	GCCAAAACCAATAAAATTACACAACAAAGAAGCGCCAT
pESC-URA-500prodGAL REEP5(G119F) F	ATGTTGAAATGCGTTTTCTTGTTGTGGTGCATGGC
pESC-URA-500prodGAL REEP5(G119F) R	CCACAACAAGAAAACGCATTTCAACATATAGTAGAAGGGGAACC
pESC-URA-500prodGAL REEP5 1/2CTH^{Y} F	AGCAAGACAGAAAAGGATGAAATTAGAGCTATTACTAAAGAGGCTAAAAAGGCTACTGT
pESC-URA-500prodGAL REEP5 1/2CTH^{Y} R	CTTTTCTGTCTTGCTAACATCTCTTAGGATATACTTCAAGAAGAATGGCCTAATAATCCTT
pESC-URA-500prodGAL REEP5 cytL^{Y} F	GACACCAACGTCCACCGATGACACACAATGGTTGACTTATTGGGTTGTTTATGG
pESC-URA-500prodGAL REEP5 cytL^{Y} R	CATCGGTGGACGTTGGTGTCTTCAAAGCCTTAATAGAAATATAAGCGGGGT
Yop1p-HA-StrepII F	GTGGAGCCACCCGCAGTTCGAAAAATAACTCGAGTAAGCTTGGTACCGC
Yop1p-HA-StrepII R	CGAACTGCGGGTGGCTCCACTCGAGAGCGTAATCTGGAACATCGTATGG
Yop1pΔnAPH/NT-TM1-HA-StrepII F	GTGGAGCCACCCGCAGTTCGAAAAATAAAAGCTTATCGATACCGTCGACCT
Yop1pΔnAPH/NT-TM1-HA-StrepII R	CGAACTGCGGGTGGCTCCACTCGAGAGCGTAATCTGGAACATCGTATGG
Yop1p(cytL-to-GS)-HA-StrepII F	GGGAGTGGAAGCAGCCAACTCTTGACCTACTGGATTGTCT
Yop1p(cytL-to-GS)-HA-StrepII R	GGCTGCTTCCACTCCCAGCAACCAACGATAAATATGCTGG
pcDNA4/TO-REEP1/5-HA-StrepII F	GAGCCACCCGCAGTTCGAAAAATAACTCGAGTCTAGAGGGCCCG
pcDNA4/TO-REEP1/5-HA-StrepII R	TTTCGAACTGCGGGTGGCTCCACTCGAGAGCGTAGTCTGGGAC
pcDNA4/TO-REEP1 1/2CTH^{Y}-HA-StrepII F	TGTTAGCAAGACAGAAAAGGATGAAATTAGAGCTTACCCATACGACGTCCCAGAC
pcDNA4/TO-REEP1 1/2CTH^{Y}-HA-StrepII R	CCTTTTCTGTCTTGCTAACATCTCTTAGGATATATTCTTTTGAAGATAGCGTGGGATGTACAA
pcDNA4/TO-REEP1 CTH^{Y}-HA-StrepII F	TGAAATTAGAGCTTCCGTCAATGAGGCTTCTAAGGCTACAGGTGCTTCTGTTCATTACCCATACGACGTCCCAGAC
pcDNA4/TO-REEP1 CTH^{Y}-HA-StrepII R	TGACGGAAGCTCTAATTTCATCCTTTTCTGTCTTGCTAACATCTCTTAGGATATATTCTTTTGAAGATAGCGTGGGATGTACAA

pcDNA4/TO-REEP1 CTH^{Y}(I70R)-HA-StrepII F	TATGAACTAAAAAGGGCATTTGTAGCCTGGCTGCT
pcDNA4/TO-REEP1 CTH^{Y}(I70R)-HA-StrepII R	GGCTACAAATGCCCTTTTTAGTTCATAATAGAATGGAAACCAACAAAGGAAGAT
pcDNA4/TO-REEP1(S3C) F	CGGATCATGGTGTGCTGGATCATCTCCAGGCTGGTG
pcDNA4/TO-REEP1(S3C) R	GGAGATGATCCAGCACACCATGATCCGAGCTCGGT
pcDNA4/TO-REEP1(S29C) F	AAGGCTGTGAAATGCAAGGACATTAAGGAATATGTCAAATGGATGA
pcDNA4/TO-REEP1(S29C) R	CTTAATGTCCTTGCATTTCACAGCCTTGTAGGAATAATACGC
pcDNA4/TO-REEP5 1/2CTH^{Y}-HA-StrepII F	ATGTTAGCAAGACAGAAAAGGATGAAATTAGAGCTTACCCATACGACGTCCCAGAC
pcDNA4/TO-REEP5 1/2CTH^{Y}-HA-StrepII R	CTTTTCTGTCTTGCTAACATCTCTTAGGATATACTTCAAGAAGAATGGCCTAATAATCCTT

Supplementary Table 3: Chemical cross-linking reagents used in this Study.

Reagent name	Chemical Structure	Catalog
Copper-o-phenanthroline	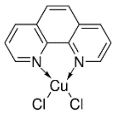	362204 (Sigma-Aldrich)
Diamide	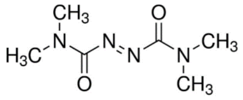	D3648 (Sigma-Aldrich)
EGS (ethylene glycol bis(succinimidyl succinate))	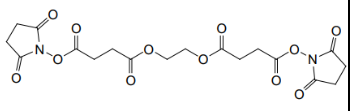	21565 (Thermo Fisher Scientific)
Maleimide PEG5K	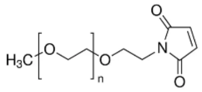	63187 (Sigma-Aldrich)
N-ethylmaleimide	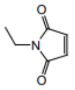	23030 (Thermo Fisher Scientific)
DL-dithiothreitol (DTT)	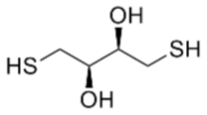	VWRC0281 (VWR)
