# Supplementary figures and images for: Oligomeric scaffolding for curvature generation by ER tubule-forming proteins
Source: Nat Commun. 2023 May 5;14:2617. doi: 10.1038/s41467-023-38294-y (PMC10162974; doi:10.1038/s41467-023-38294-y)

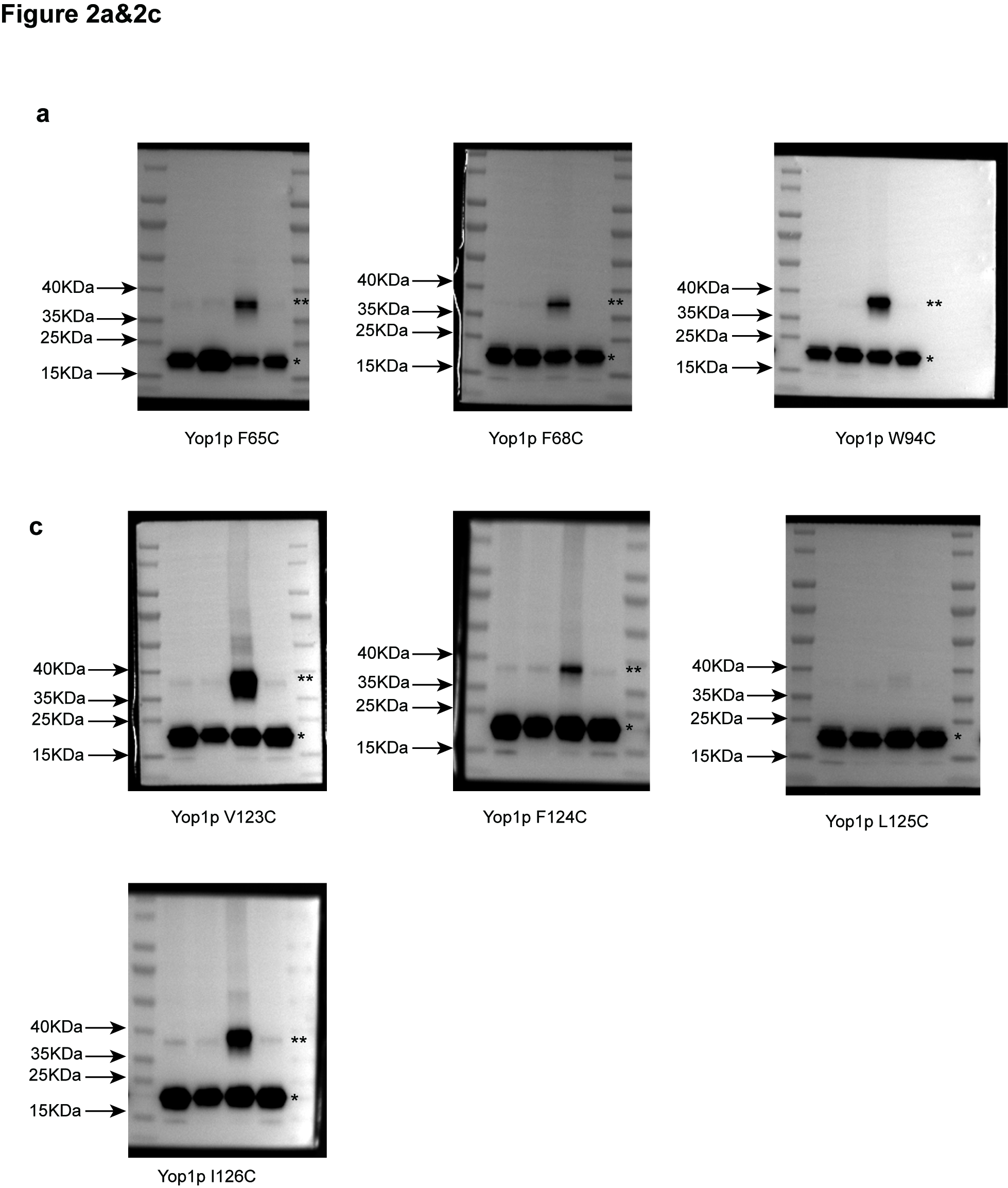

Supplement: Supplementary file 4 — Source Data [file 41467_2023_38294_MOESM4_ESM.zip › Hu Source Data/SourceData Fig 2.tif]

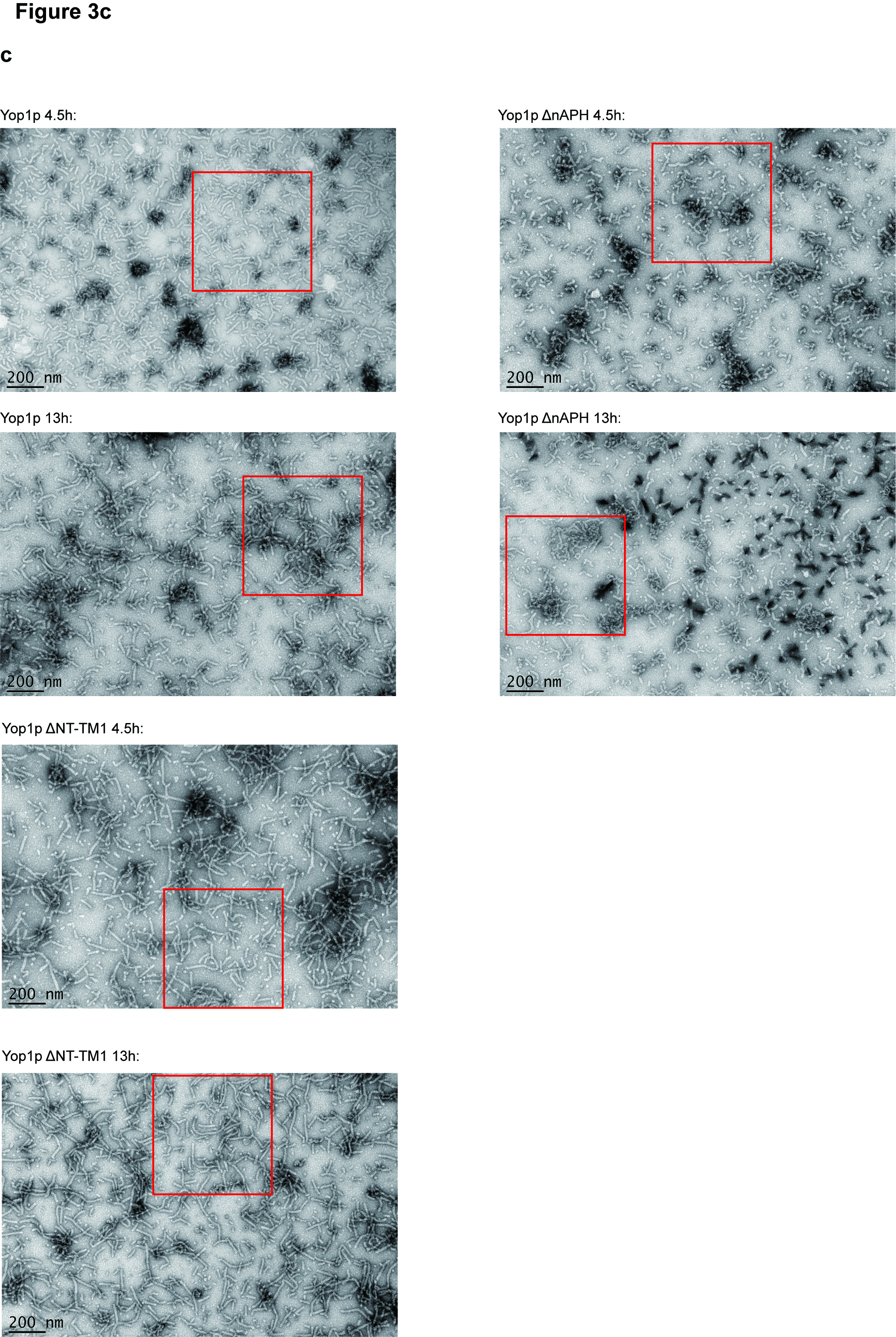

Supplement: Supplementary file 4 — Source Data [file 41467_2023_38294_MOESM4_ESM.zip › Hu Source Data/SourceData Fig 3.tif]

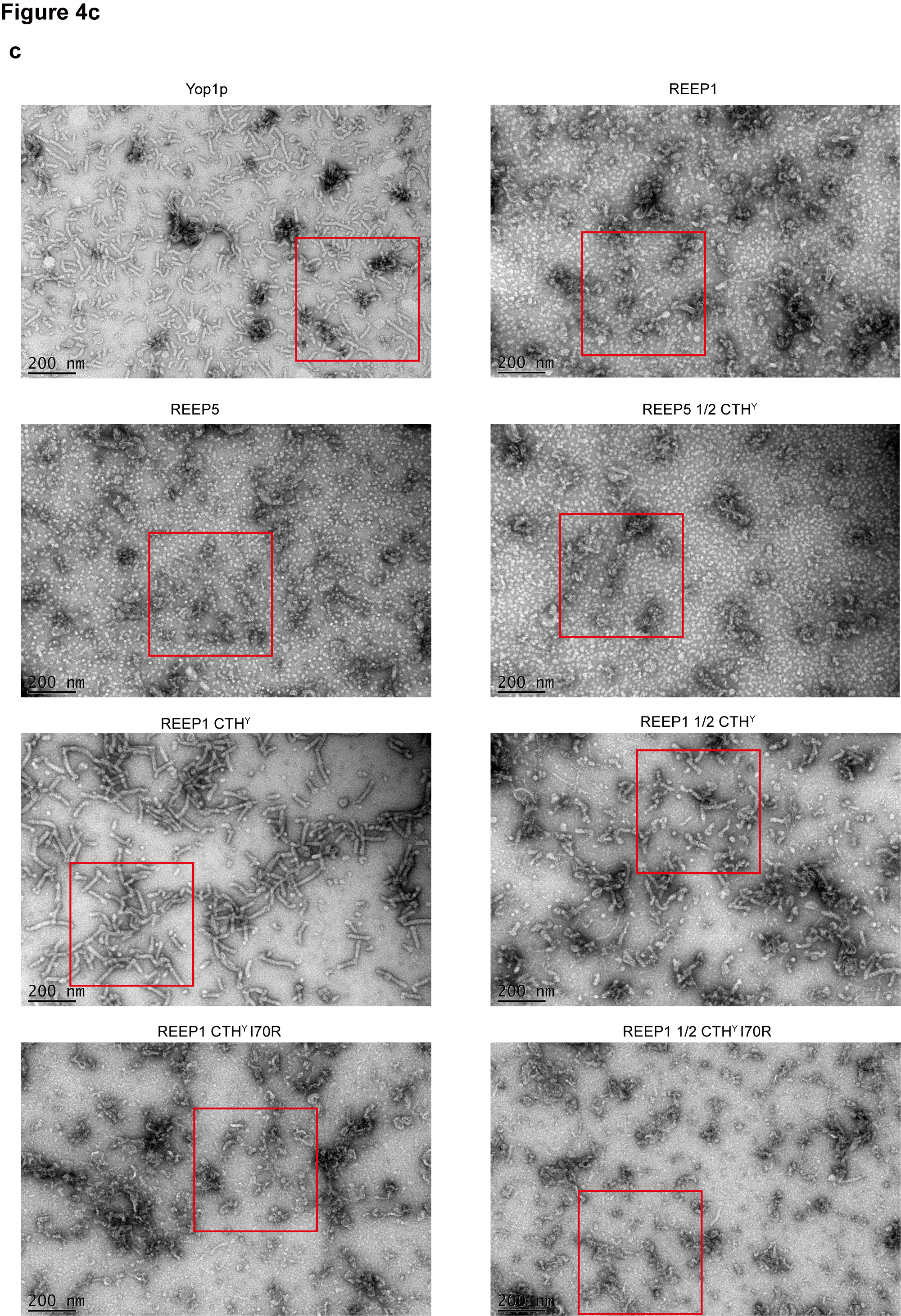

Supplement: Supplementary file 4 — Source Data [file 41467_2023_38294_MOESM4_ESM.zip › Hu Source Data/SourceData Fig4.tif]

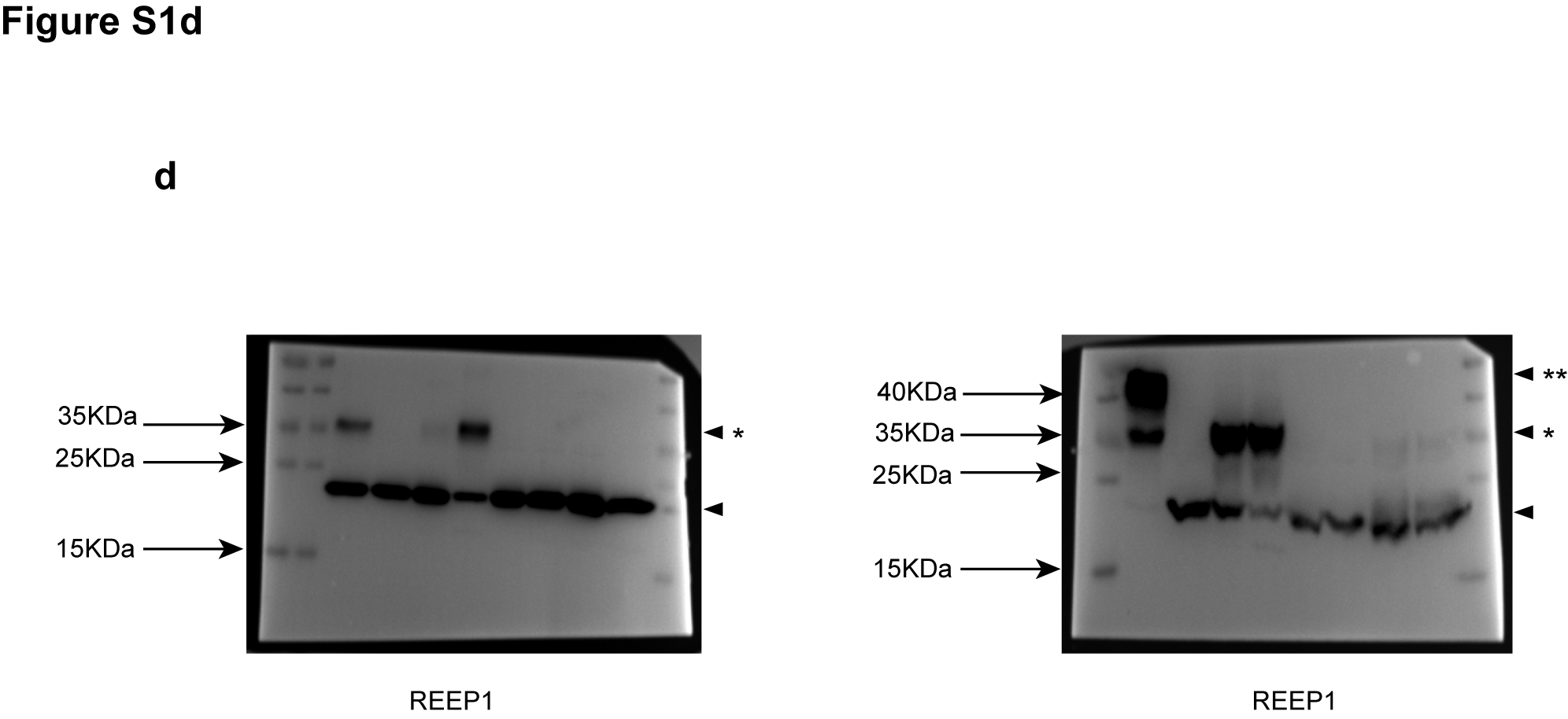

Supplement: Supplementary file 4 — Source Data [file 41467_2023_38294_MOESM4_ESM.zip › Hu Source Data/SourceData Sup Fig1.tif]

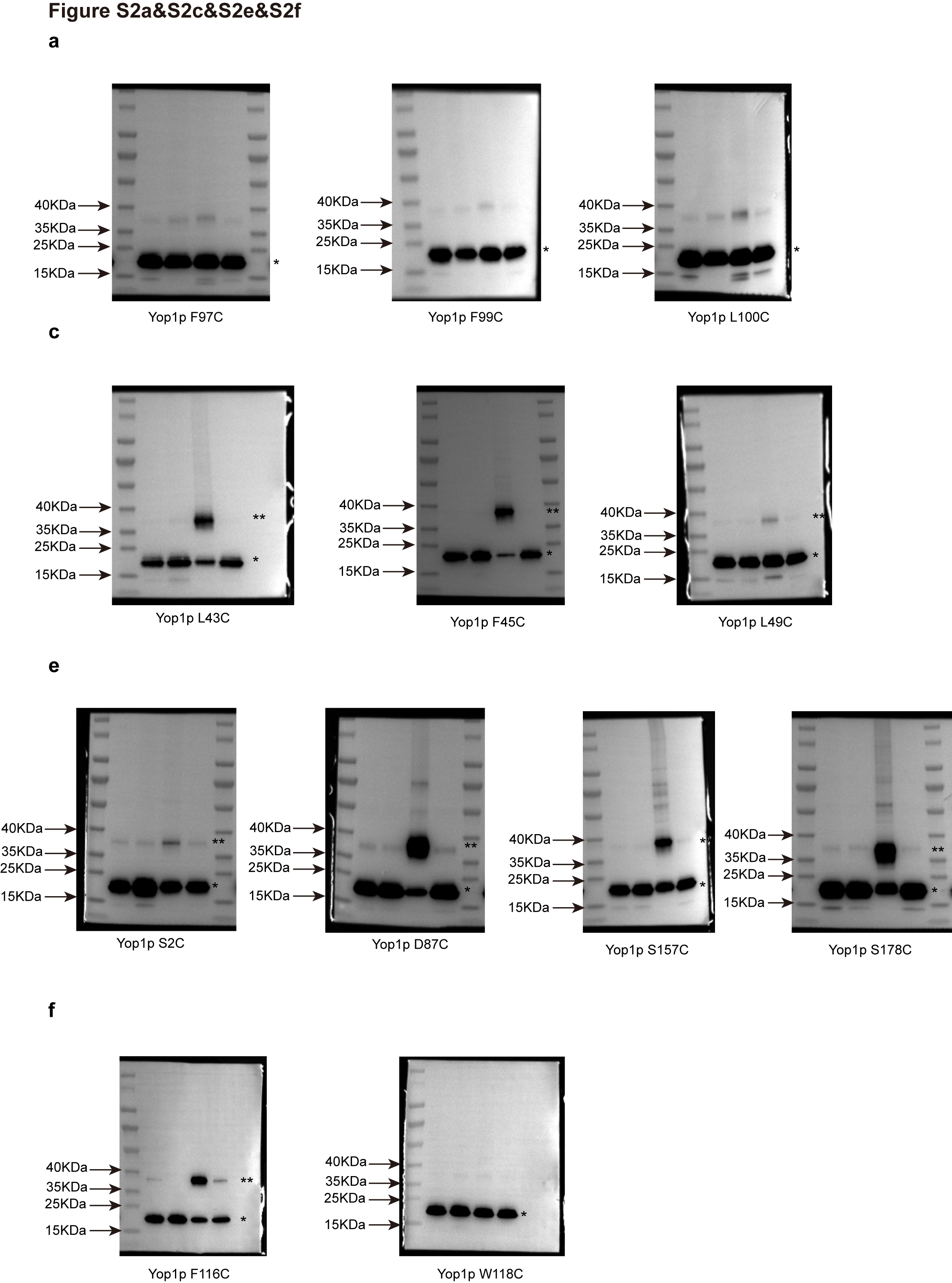

Supplement: Supplementary file 4 — Source Data [file 41467_2023_38294_MOESM4_ESM.zip › Hu Source Data/SourceData Sup Fig2.tif]

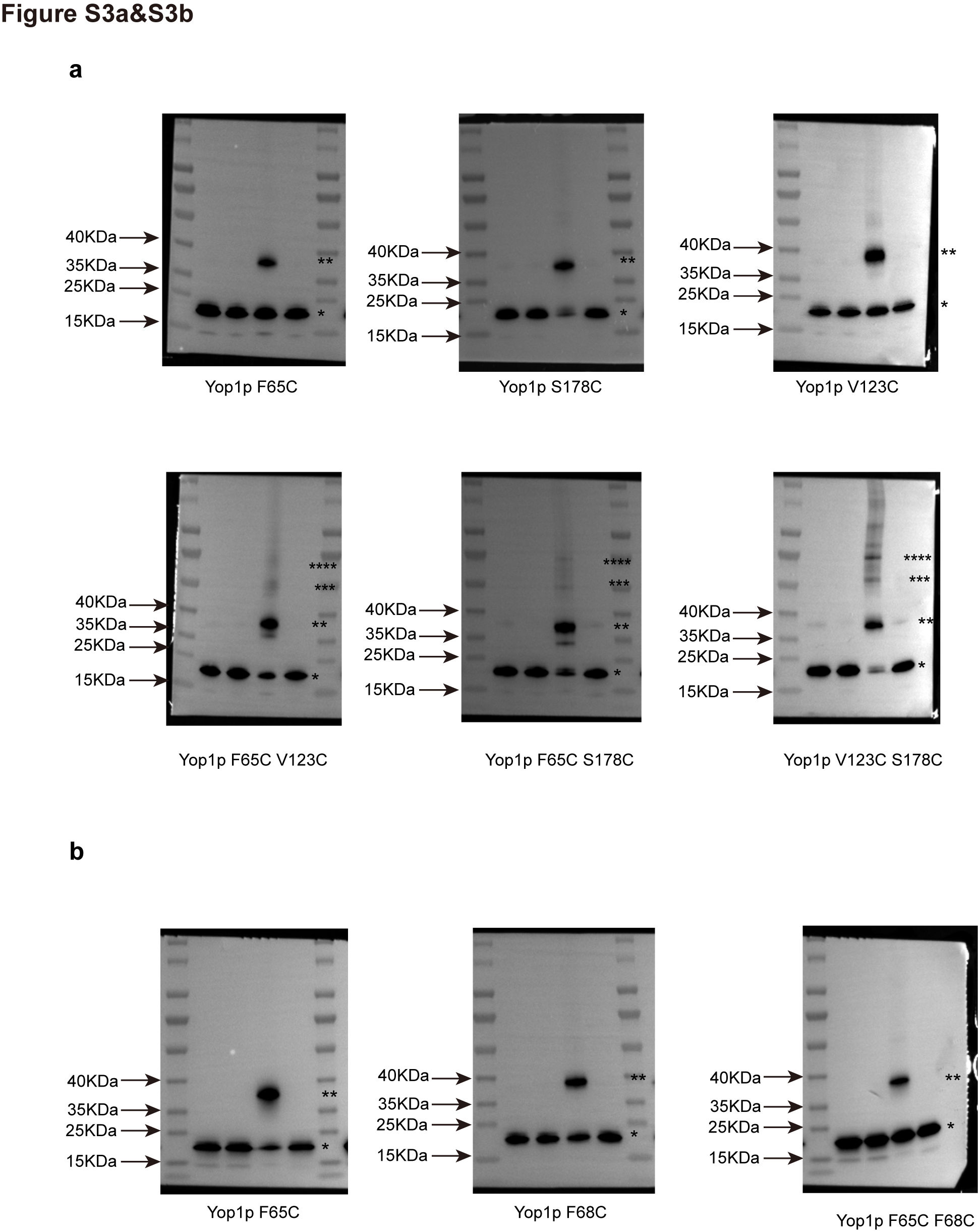

Supplement: Supplementary file 4 — Source Data [file 41467_2023_38294_MOESM4_ESM.zip › Hu Source Data/SourceData Sup Fig3.tif]

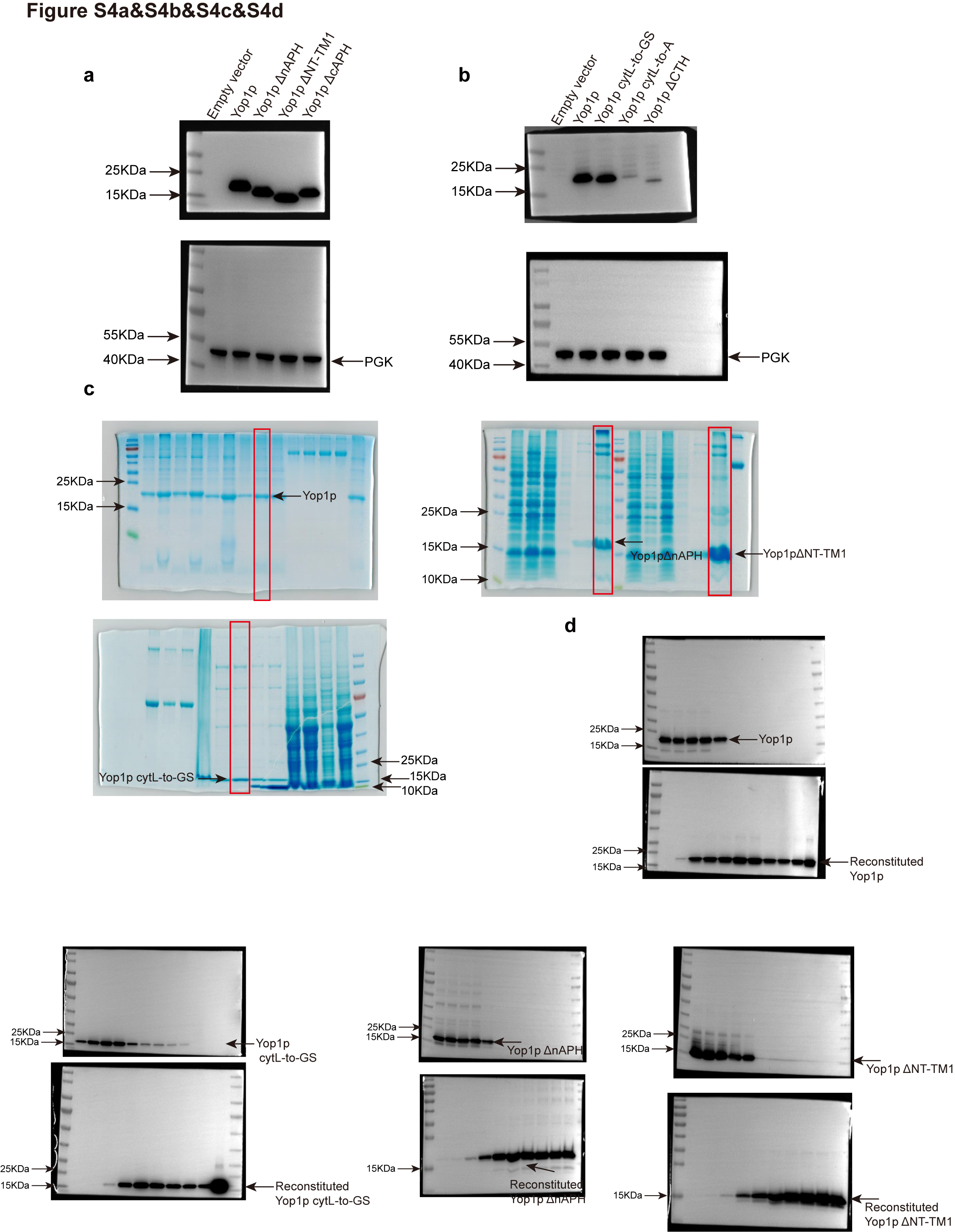

Supplement: Supplementary file 4 — Source Data [file 41467_2023_38294_MOESM4_ESM.zip › Hu Source Data/SourceData Sup Fig4-2.tif]

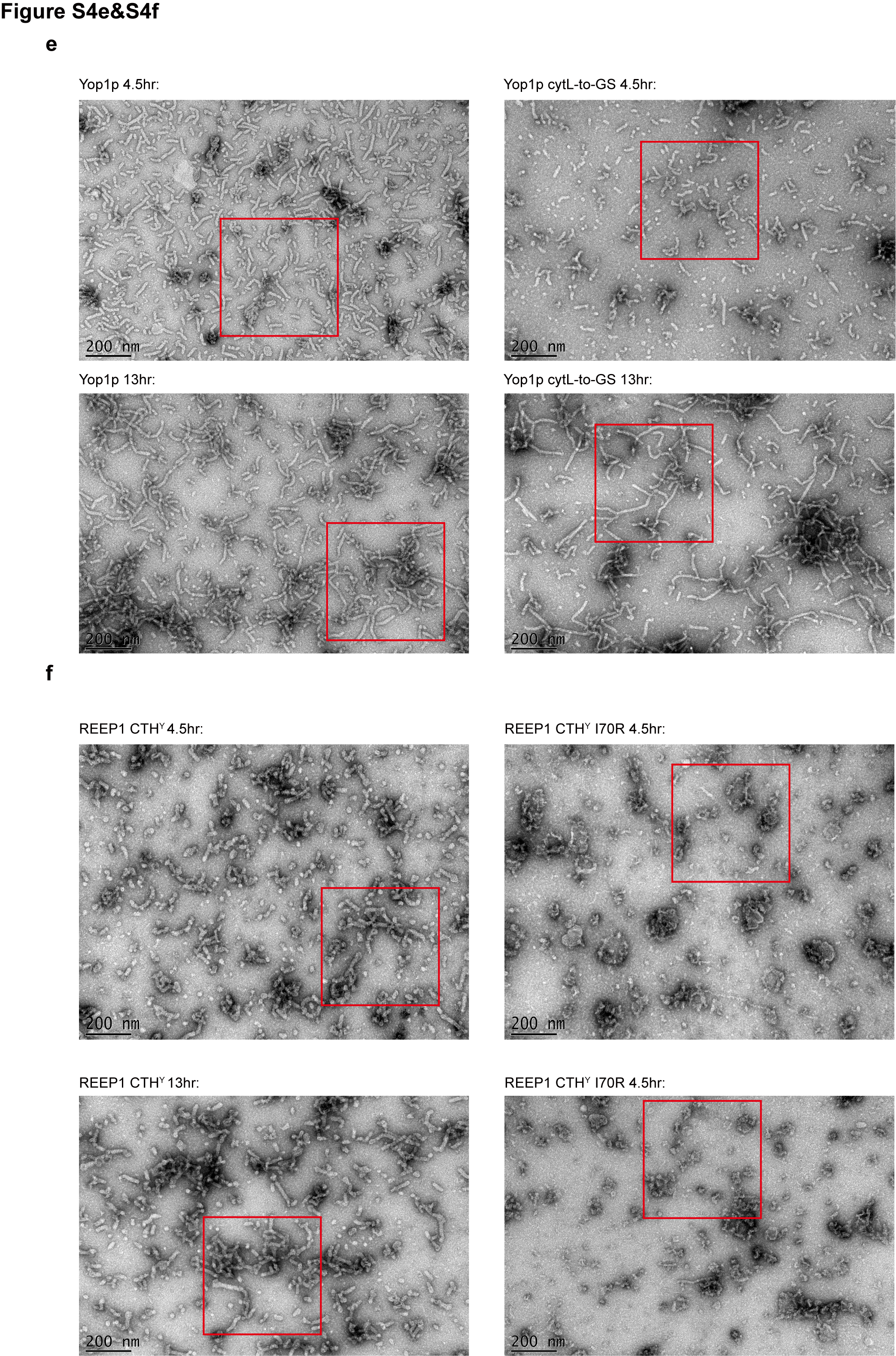

Supplement: Supplementary file 4 — Source Data [file 41467_2023_38294_MOESM4_ESM.zip › Hu Source Data/SourceData Sup Fig4.tif]

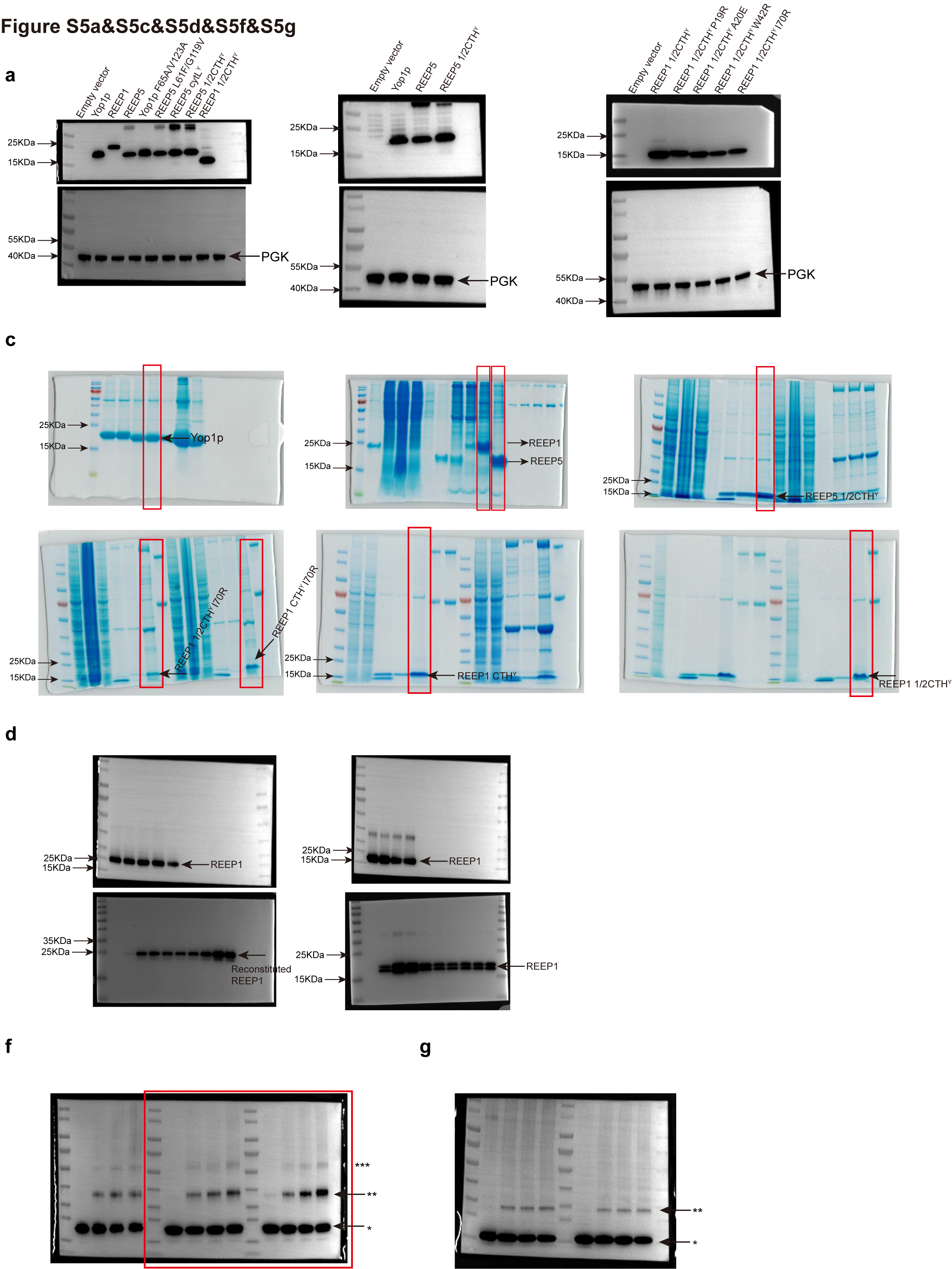

Supplement: Supplementary file 4 — Source Data [file 41467_2023_38294_MOESM4_ESM.zip › Hu Source Data/SourceData Sup Fig5.tif]
