# Supplementary material for: Publisher Correction: Oligomeric scaffolding for curvature generation by ER tubule-forming proteins
Source: Nat Commun. 2023 Jun 13;14:3483. doi: 10.1038/s41467-023-39182-1 (PMC10264451; doi:10.1038/s41467-023-39182-1)
Supplement: Supplementary file 1 — Reporting Summary [file 41467_2023_39182_MOESM1_ESM.pdf]

nature portfolio

Corresponding author(s): Junjie Hu

Last updated by author(s): Mar 25, 2023

Reporting Summary

Nature Portfolio wishes to improve the reproducibility of the work that we publish. This form provides structure for consistency and transparency in reporting. For further information on Nature Portfolio policies, see our Editorial Policies and the Editorial Policy Checklist.

Statistics

For all statistical analyses, confirm that the following items are present in the figure legend, table legend, main text, or Methods section.

n/a	Confirmed	
☐	☒	The exact sample size (*n*) for each experimental group/condition, given as a discrete number and unit of measurement
☐	☒	A statement on whether measurements were taken from distinct samples or whether the same sample was measured repeatedly
☐	☒	The statistical test(s) used AND whether they are one- or two-sided<br>*Only common tests should be described solely by name; describe more complex techniques in the Methods section.*
☒	☐	A description of all covariates tested
☒	☐	A description of any assumptions or corrections, such as tests of normality and adjustment for multiple comparisons
☐	☒	A full description of the statistical parameters including central tendency (e.g. means) or other basic estimates (e.g. regression coefficient) AND variation (e.g. standard deviation) or associated estimates of uncertainty (e.g. confidence intervals)
☐	☒	For null hypothesis testing, the test statistic (e.g. *F*, *t*, *r*) with confidence intervals, effect sizes, degrees of freedom and *P* value noted<br>*Give P values as exact values whenever suitable.*
☒	☐	For Bayesian analysis, information on the choice of priors and Markov chain Monte Carlo settings
☒	☐	For hierarchical and complex designs, identification of the appropriate level for tests and full reporting of outcomes
☒	☐	Estimates of effect sizes (e.g. Cohen's *d*, Pearson's *r*), indicating how they were calculated

Our web collection on statistics for biologists contains articles on many of the points above.

Software and code

Policy information about availability of computer code

Data collection	The amino acid sequence of proteins were downloaded from the Uniport website.<br>The REEP structures were predicted by AlphaFold and RoseTTAFold online server.<br>The Negative stain EM images were collected by a Hitachi HT7700 electron microscope.<br>The fluorescent microscopy images were collected by the DeltaVision OMX V3 imaging system (Cytiva, GE Healthcare).<br>Tanon 4600SF Chemiluminescence image analysis system.
Data analysis	ImageJ (version 1.53a, US NIH); GraphPad Prism software (version 8, La jolla,CA); PyMOL(version 2.5.3, Schrödinger, LLC.)

For manuscripts utilizing custom algorithms or software that are central to the research but not yet described in published literature, software must be made available to editors and reviewers. We strongly encourage code deposition in a community repository (e.g. GitHub). See the Nature Portfolio guidelines for submitting code & software for further information.

Data

Policy information about availability of data

All manuscripts must include a data availability statement. This statement should provide the following information, where applicable:

- Accession codes, unique identifiers, or web links for publicly available datasets
- A description of any restrictions on data availability
- For clinical datasets or third party data, please ensure that the statement adheres to our policy

All data generated or analyzed during this study are included in this published article and its supplementary information file and source Data file.

Human research participants

Policy information about studies involving human research participants and Sex and Gender in Research.

Reporting on sex and gender	N/A
Population characteristics	N/A
Recruitment	N/A
Ethics oversight	N/A

Note that full information on the approval of the study protocol must also be provided in the manuscript.

Field-specific reporting

Please select the one below that is the best fit for your research. If you are not sure, read the appropriate sections before making your selection.

☒ Life sciences ☐ Behavioural & social sciences ☐ Ecological, evolutionary & environmental sciences

For a reference copy of the document with all sections, see nature.com/documents/nr-reporting-summary-flat.pdf

Life sciences study design

All studies must disclose on these points even when the disclosure is negative.

Sample size	For negative stain-EM images, 50 images were taken for each sample and experiments were performed 2 times;for fluorescent microscopy images, at least 100 images were taken for each sample were used for quantification analysis and experiments were performed 3 times. Samples sizes were chosen to be 3 for in vitro biochemical analysis to achieve statistical significance.
Data exclusions	No data were excluded from the analyses.
Replication	All experiments were repeated three times, except that the in vitro tubule formation assay were repeated twice, and all attempts at replication were successful.
Randomization	The expressed yeast clones and Image fields were selected randomly; Expi293F cells were parallel cultured and randomly assigned to transfection with the different constructs.
Blinding	During negative stain-EM, fluorescent microscopy data collection and statistical analyses, the fields of view were chosen on a random basis, and the ER morphology were defined by different operators, preventing potentially biased selection for desired phenotypes.

Reporting for specific materials, systems and methods

We require information from authors about some types of materials, experimental systems and methods used in many studies. Here, indicate whether each material, system or method listed is relevant to your study. If you are not sure if a list item applies to your research, read the appropriate section before selecting a response.

Materials & experimental systems

n/a	Involved in the study
☐	☒ Antibodies
☐	☒ Eukaryotic cell lines
☒	☐ Palaeontology and archaeology
☒	☐ Animals and other organisms
☒	☐ Clinical data
☒	☐ Dual use research of concern

Methods

n/a	Involved in the study
☒	☐ ChIP-seq
☒	☐ Flow cytometry
☒	☐ MRI-based neuroimaging

Antibodies

Antibodies used	Primary antibodies: anti-HA (C29F4, 1:2000, CST, Cat. No: 3724S, Lot: 10); anti-PGK1 (22C5D8, 1:2000, Abcam, Cat. No: ab113687, Lot:GR3373682-13)<br>Secondary antibodies: anti-Mouse IgG-HRP (1:20000, KPL, Cat. No: 5450-0011 Lot: 10473846); anti-Rabbit IgG-HRP (1:20000, KPL, Cat. No: 5450-0010 Lot: 10437708);
Validation	All antibodies used in this study are commercially available and have been validated by the manufacturers accordingly.<br>anti-HA (C29F4, 1:2000, CST, Cat. No: 3724S, Lot: 10), Host: Rabbit; Reactivity: All Species Expected; Application:IF,WB,CHIP,IP,IHC; antibody website: https://www.cellsignal.com/products/primary-antibodies/ha-tag-c29f4-rabbit-mab/3724<br>anti-PGK1 (22C5D8, 1:2000, Abcam, Cat. No: ab113687, Lot:GR3373682-13), Host: Mouse; Reactivity: Saccharomyces cerevisiae; Application: WB; antibody website: https://www.abcam.com/pgk1-antibody-22c5d8-ab113687.html

Eukaryotic cell lines

Policy information about cell lines and Sex and Gender in Research

Cell line source(s)	COS-7 cells are from ATCC. Expi293F cells were purchase from Thermo Fisher Scientific.
Authentication	COS-7 cells were obtained from ATCC and have been authenticated by STR profiling. Expi293F cells were purchase from Thermo Fisher Scientific and have been authenticated by STR profiling.
Mycoplasma contamination	These two cell lines are tested negative for mycoplasma contamination.
Commonly misidentified lines<br>(See ICLAC register)	No commonly misidentified cell lines were used.
