# Supplementary figures and images for: Publisher Correction: Oligomeric scaffolding for curvature generation by ER tubule-forming proteins
Source: Nat Commun. 2023 Jun 13;14:3483. doi: 10.1038/s41467-023-39182-1 (PMC10264451; doi:10.1038/s41467-023-39182-1)

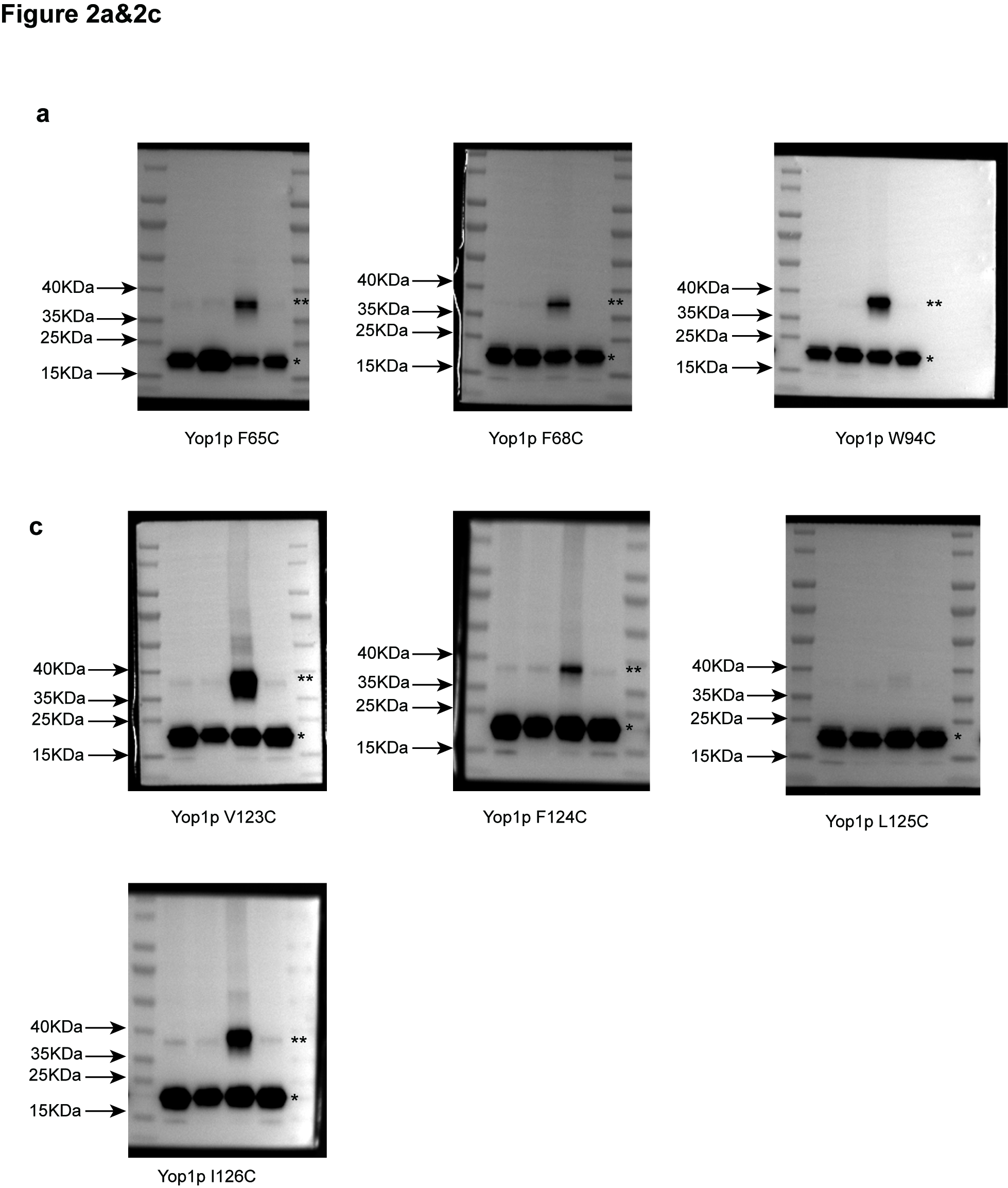

Supplement: Supplementary file 2 — Source Data [file 41467_2023_39182_MOESM2_ESM.zip › Hu Source Data/SourceData Fig 2.tif]

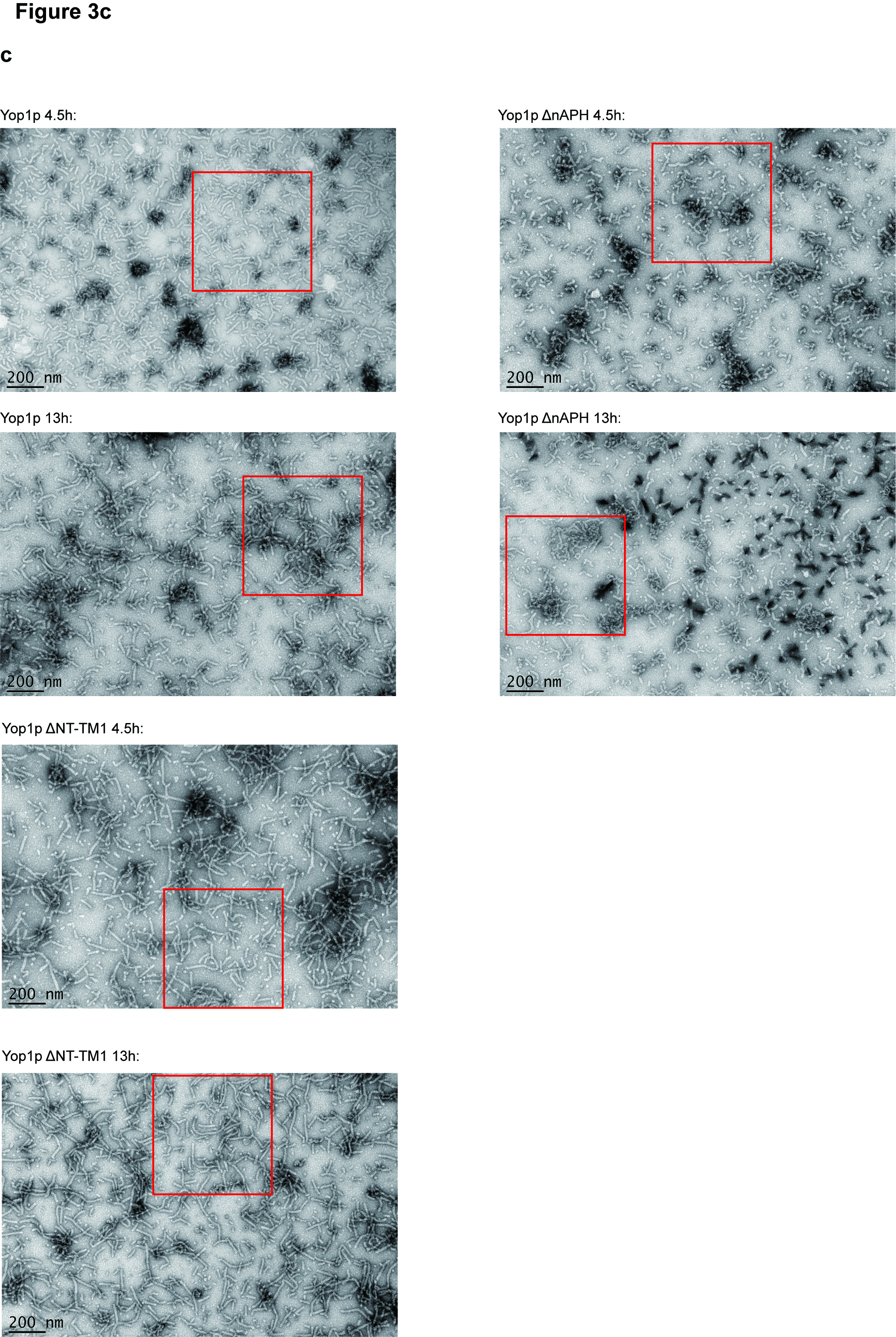

Supplement: Supplementary file 2 — Source Data [file 41467_2023_39182_MOESM2_ESM.zip › Hu Source Data/SourceData Fig 3.tif]

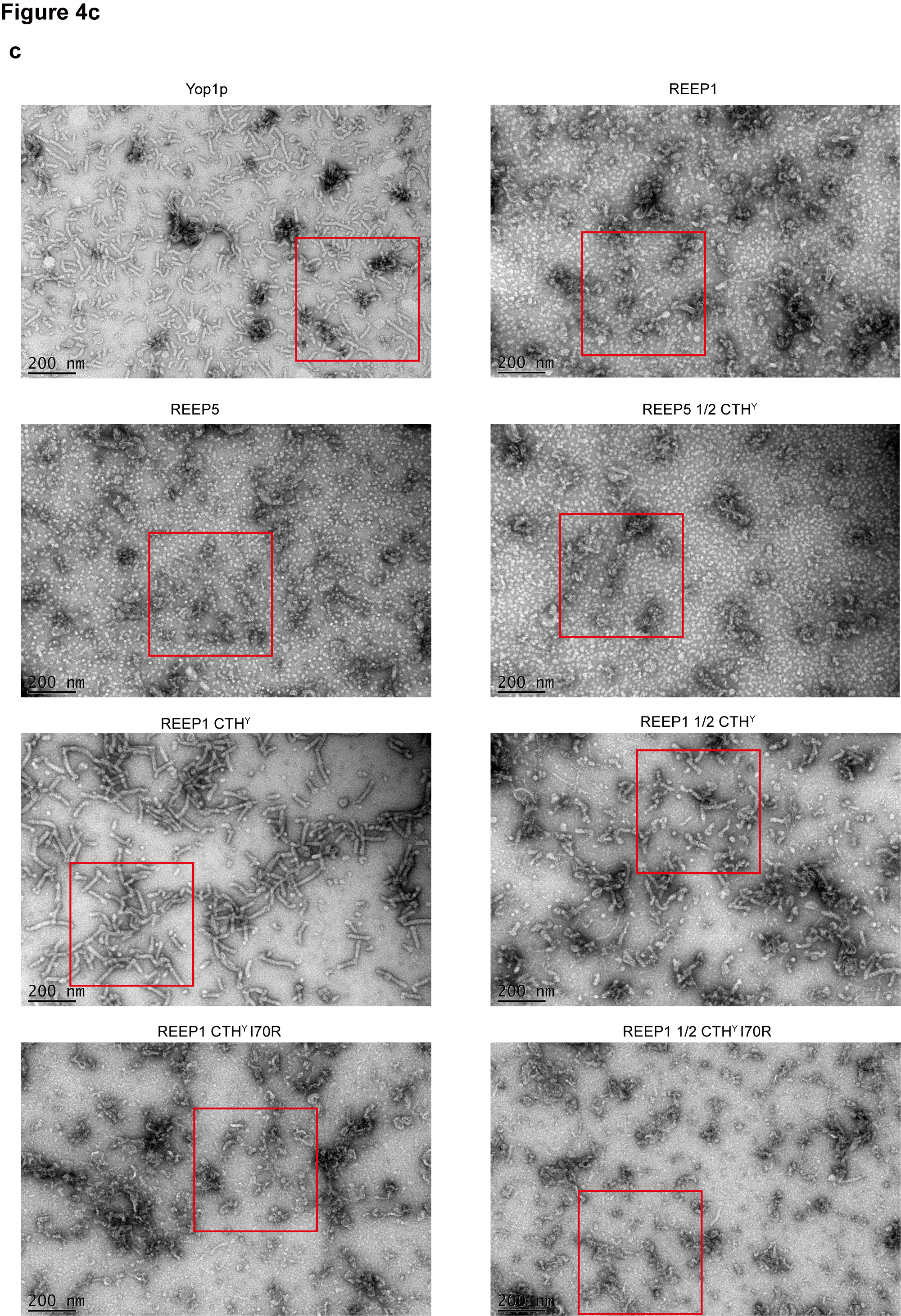

Supplement: Supplementary file 2 — Source Data [file 41467_2023_39182_MOESM2_ESM.zip › Hu Source Data/SourceData Fig4.tif]

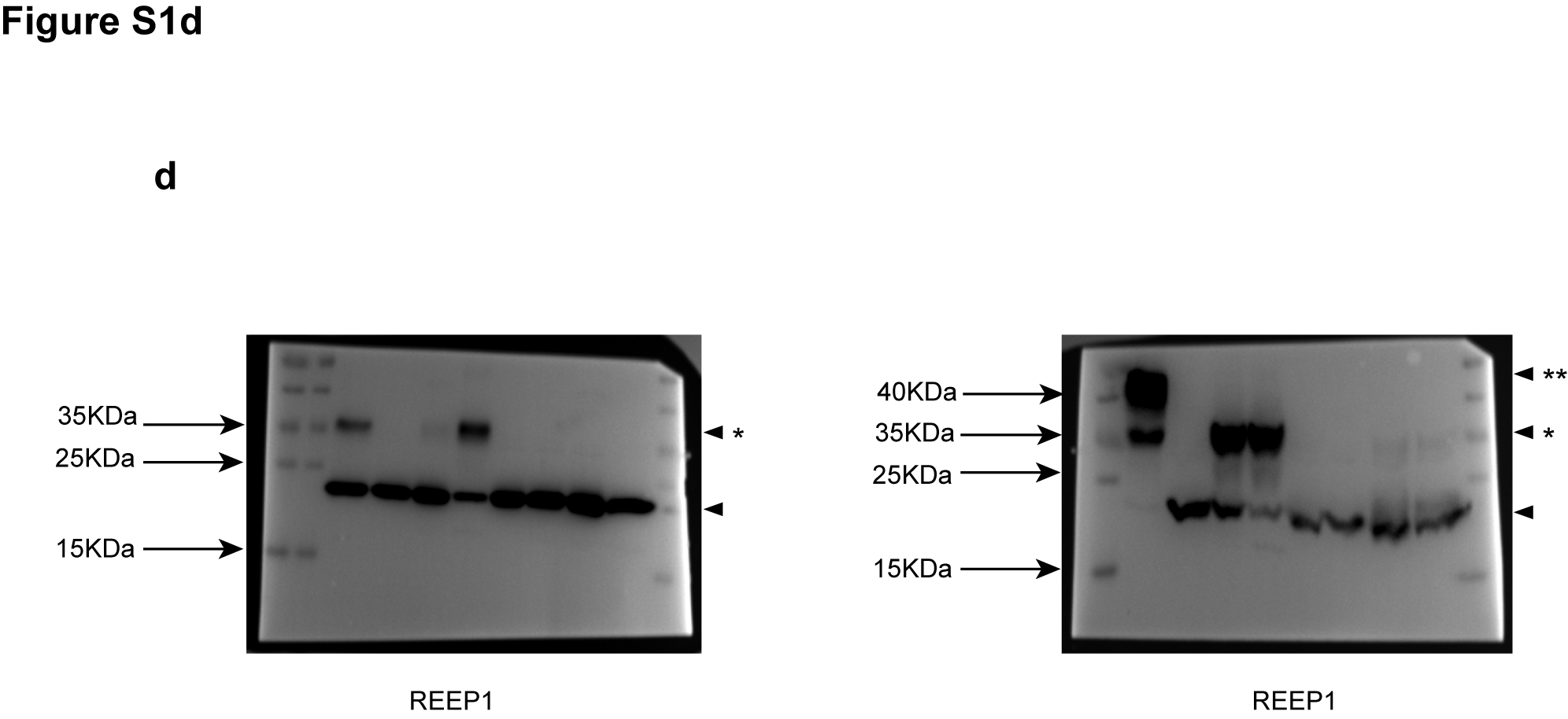

Supplement: Supplementary file 2 — Source Data [file 41467_2023_39182_MOESM2_ESM.zip › Hu Source Data/SourceData Sup Fig1.tif]

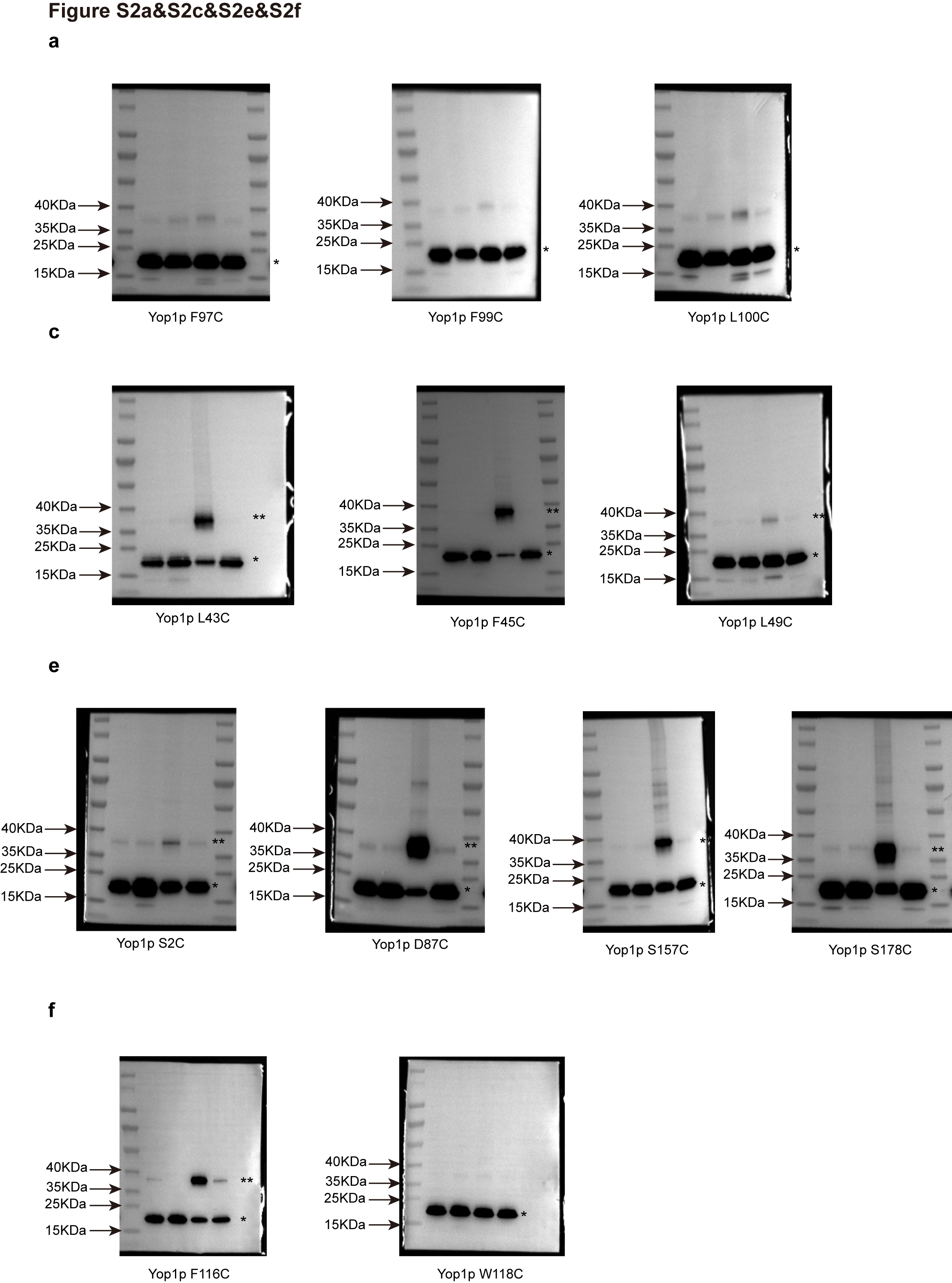

Supplement: Supplementary file 2 — Source Data [file 41467_2023_39182_MOESM2_ESM.zip › Hu Source Data/SourceData Sup Fig2.tif]

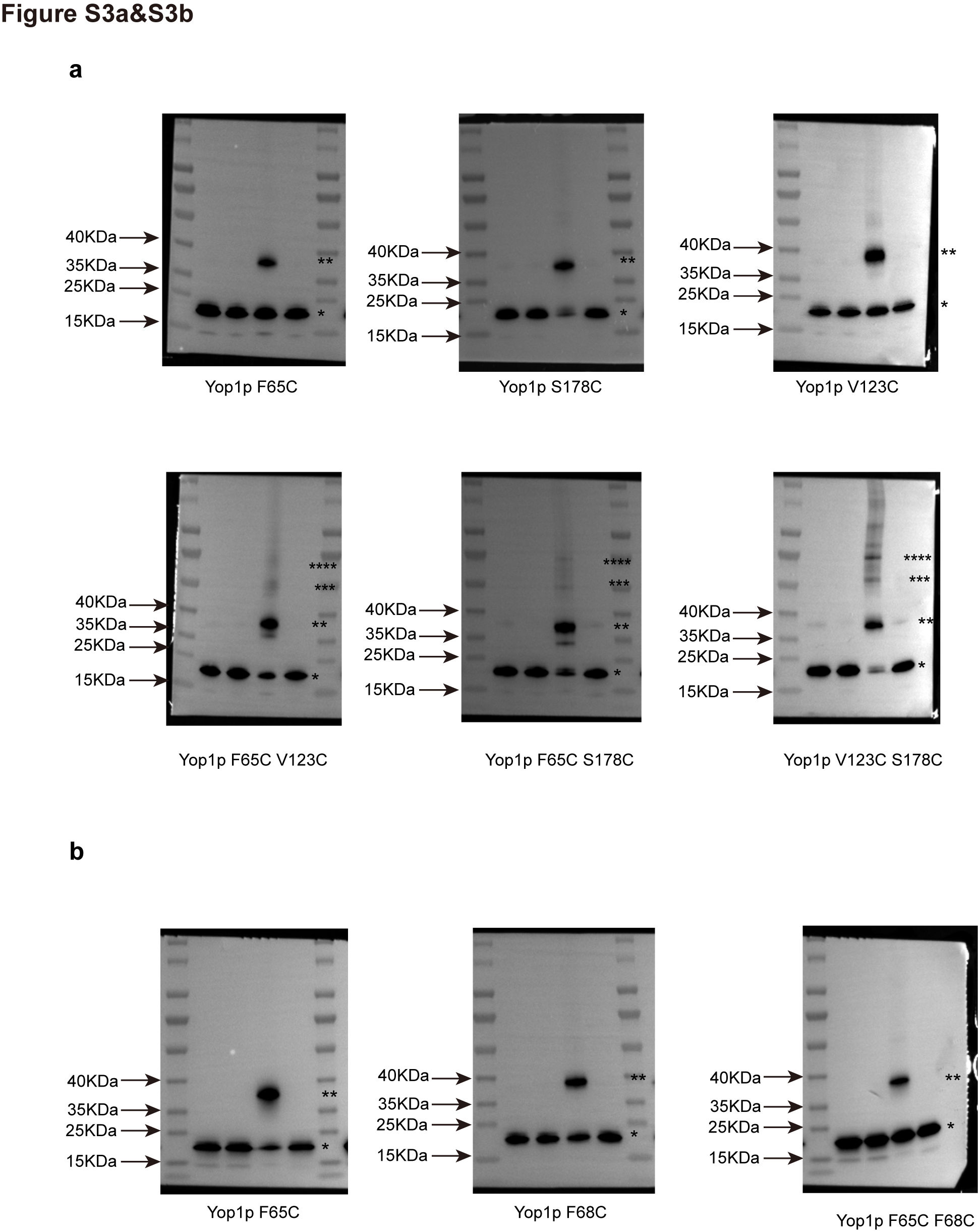

Supplement: Supplementary file 2 — Source Data [file 41467_2023_39182_MOESM2_ESM.zip › Hu Source Data/SourceData Sup Fig3.tif]

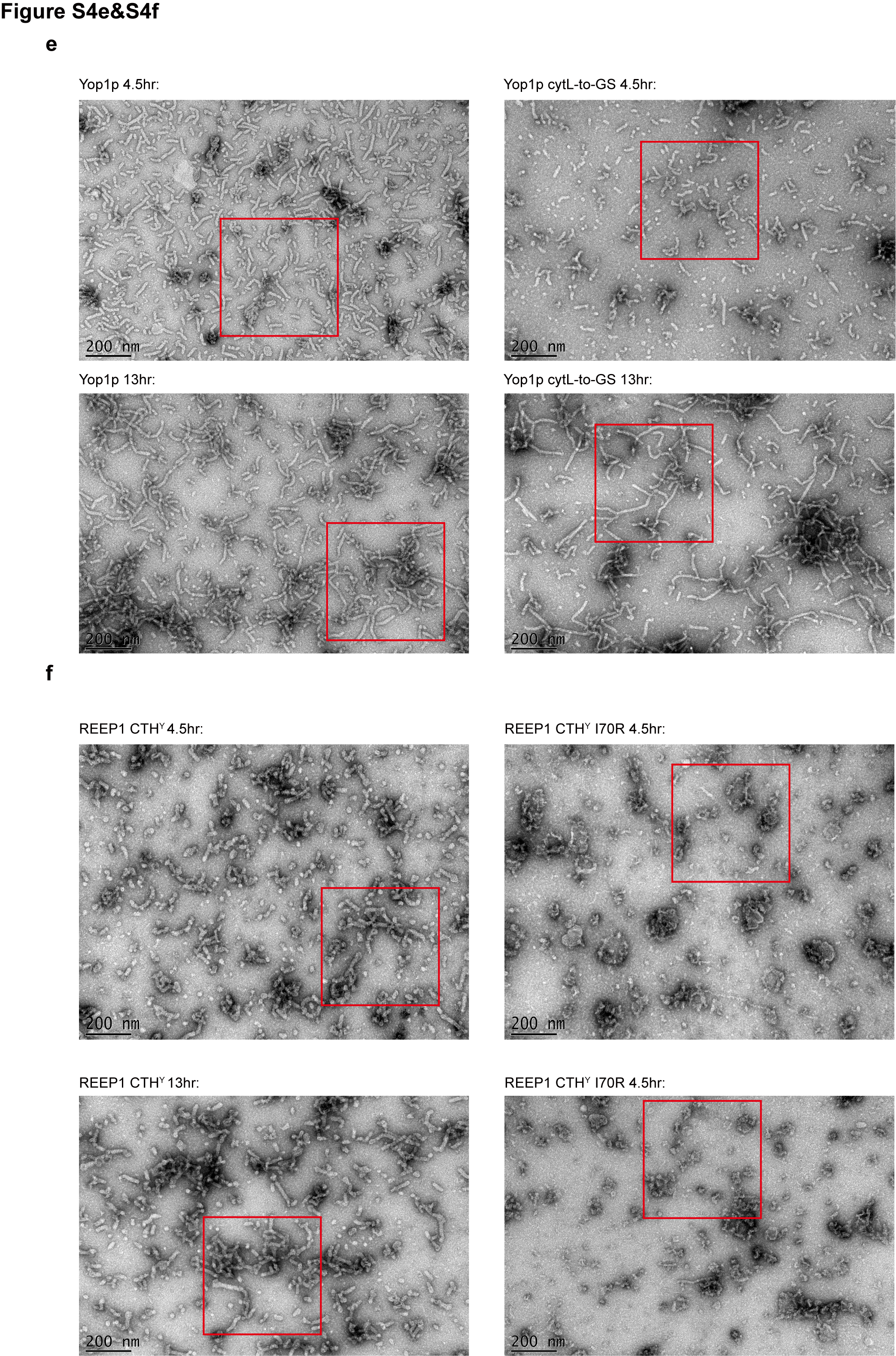

Supplement: Supplementary file 2 — Source Data [file 41467_2023_39182_MOESM2_ESM.zip › Hu Source Data/SourceData Sup Fig4.tif]

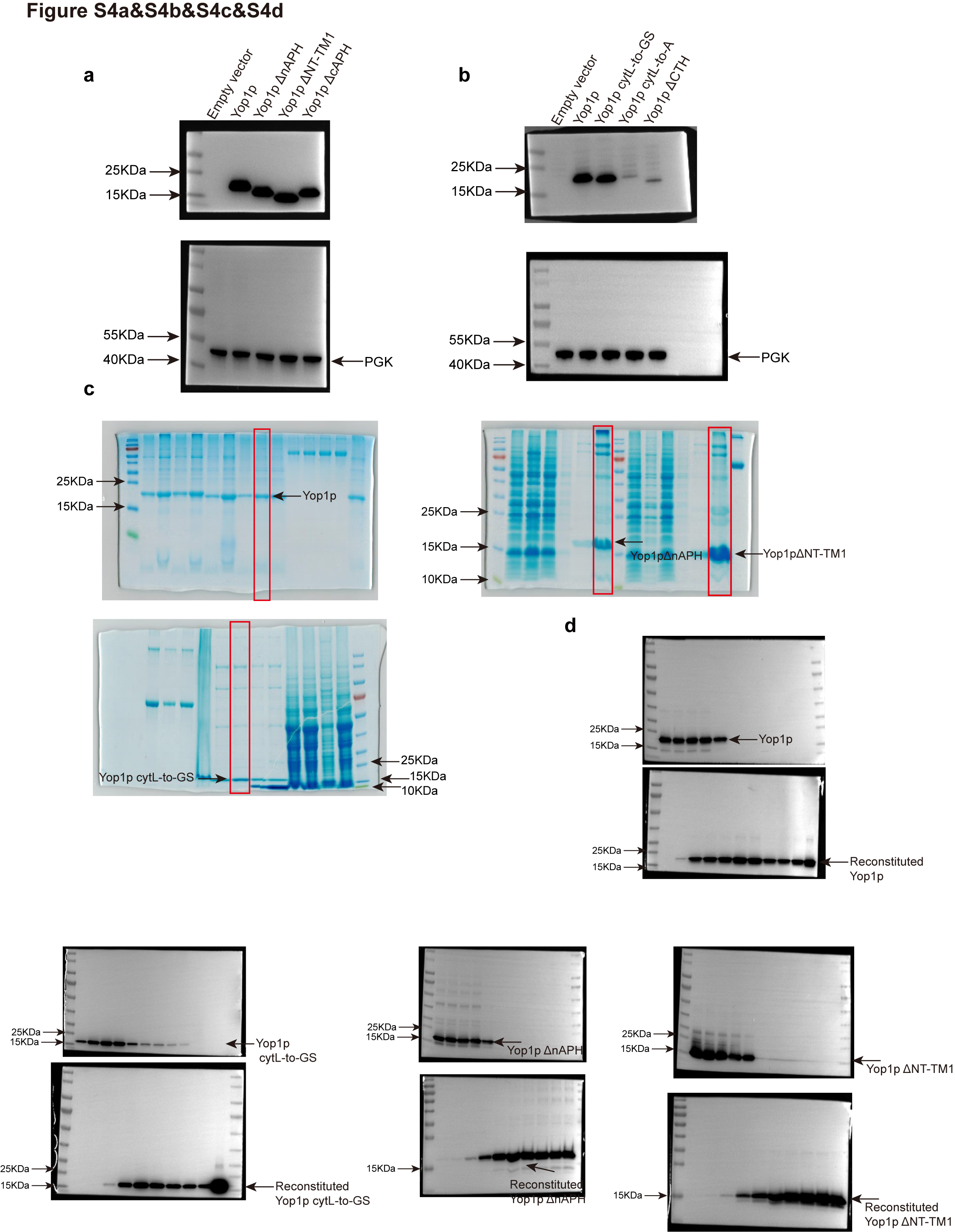

Supplement: Supplementary file 2 — Source Data [file 41467_2023_39182_MOESM2_ESM.zip › Hu Source Data/SourceData Sup Fig4-2.tif]

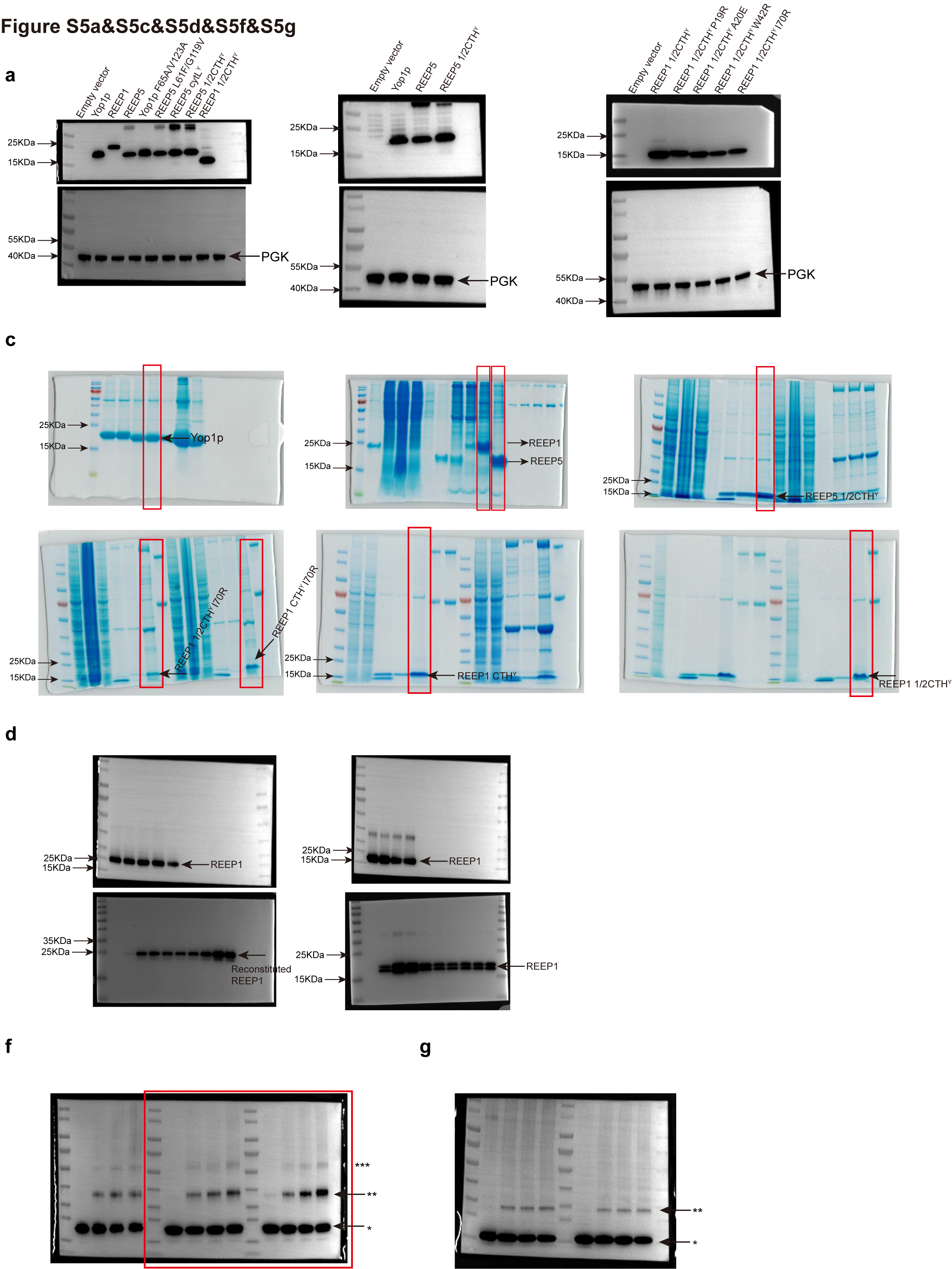

Supplement: Supplementary file 2 — Source Data [file 41467_2023_39182_MOESM2_ESM.zip › Hu Source Data/SourceData Sup Fig5.tif]
